# Biomimetic composite hydrogel promotes new bone formation in rat bone defects through regulation of miR-19b-3p/WWP1 axis by loaded extracellular vesicles

**DOI:** 10.1186/s12951-023-02201-w

**Published:** 2023-11-30

**Authors:** Rongkang Guo, Chaohan Wu, Fan Liu, Tianhua Dong, Tao Zhang

**Affiliations:** Department of Emergency Trauma Center, The Third Hospital of Hebei Medical University, 139 Ziqiang Road, Shijiazhuang, 050051 Hebei Province People’s Republic of China

**Keywords:** Biomimetic composite hydrogel, Extracellular vesicles, miR-19b-3p, WWP1, Bone defect repair, BMSCs, Osteogenic differentiation, Rat model

## Abstract

**Objective:**

This study aims to investigate the mechanism by which biomimetic composite hydrogels loaded with bone marrow mesenchymal stem cells (BMSCs) derived microRNA-19b-3p/WWP1 axis through extracellular vesicles (EVs) affect the new bone formation in rat bone defects.

**Methods:**

First, synthesize the bionic composite hydrogel Gel-OCS/MBGN. Characterize it through field-emission scanning electron microscopy (FE-SEM), X-ray diffraction (XRD), and FTIR. Then, conduct performance tests such as rheology, dynamic mechanical analysis, in vitro mineralization, and degradation. Rat BMSCs were selected for in vitro cell experiments, and EVs derived from BMSCs were obtained by differential centrifugation. The EVs were loaded onto Gel-OCS/MBGN to obtain Gel-OCS/MBGN@EVs hydrogel. Cell viability and proliferation were detected by live/dead cell staining and CCK-8 assay, respectively. ALP and ARS staining was used to evaluate the osteogenic differentiation of BMSCs. Differential gene expression analysis of osteogenic differentiation was performed using high-throughput sequencing. TargetScan database predicted the binding site between miR-19b-3p and WWP1, and a dual-luciferase reporter assay was performed to confirm the targeting binding site. A rat bone defect model was established, and new bone formation was evaluated by Micro-CT, H&E staining, and Masson's trichrome staining. Immunofluorescence staining and immunohistochemistry were used to detect the expression levels of osteogenic-related factors in rat BMSCs. RT-qPCR and Western blot were used to detect the expression levels of genes and proteins in tissues and cells.

**Result:**

Gel-OCS/MBGN was successfully constructed and loaded with EVs, resulting in Gel-OCS/MBGN@EVs. The in vitro drug release experiment results show that Gel-OCS/MBGN could sustainably release EVs. Further experiments have shown that Gel-OCS/MBGN@EVs could significantly promote the differentiation of BMSCs into osteoblasts. Experiments have shown that WWP1 is a key factor in osteogenic differentiation and is regulated by miR-19b-3p. EVs promote osteogenic differentiation by suppressing WWP1 expression through the transmission of miR-19b-3p. In vivo animal experiments have demonstrated that Gel-OCS/MBGN@EVs significantly promote bone repair in rats with bone defects by regulating the miR-19b-3p/WWP1 signaling axis.

**Conclusion:**

Functional Gel-OCS/MBGN@EVs were obtained by constructing Gel-OCS/MBGN and loading EVs onto it. EVs could deliver miR-19b-3p to BMSCs, inhibit the expression of WWP1, and promote the osteogenic differentiation of BMSCs, ultimately promoting bone regeneration in rats with bone defects.

**Graphical Abstract:**

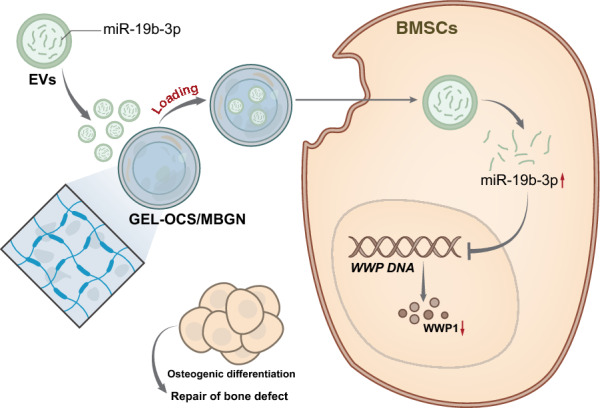

**Supplementary Information:**

The online version contains supplementary material available at 10.1186/s12951-023-02201-w.

## Introduction

The use of bone transplantation utilizes the principle of "tension-stress law". During the puncture process outside the body, the steel nail is fixed on the bone in the fracture area, and the bone is gradually lengthened by bone cutting [1]. During this process, both elongation and compression regions benefit the formation of calli until the bone defect is healed [2, 3]. Although bone transplantation surgery has the advantages of simple operation, small trauma, and fast bone repair, its surgical operation is complex, and the incidence of complications is high [1, 4].

With the development of bone tissue engineering (BTE), increasing evidence suggests that extracellular vesicles (EVs) derived from mesenchymal stem cells, especially exosomes, have excellent bone regenerative potential [5, 6]. However, current research on the repair of bone defects by MSC-EVs is still in its infancy [5]. Biomaterials with biological activity, such as scaffolds combined with MSCs and their secreted factors EVs, have been widely used in bone defect repair [7]. Research has shown that biomaterials such as hydrogels or scaffolds loaded with mesenchymal stem cells-derived extracellular vesicles could significantly promote osteogenesis and angiogenesis, thereby effectively repairing bone defects [8, 9]. Chondroitin sulfate (CS) is a glycosaminoglycan found in the non-collagen extracellular matrix (ECM) of human bones. Research has shown that CS improves bone regeneration and enhances the arrangement of growth factors involved in bone regeneration. Mesoporous bioactive glass nanoparticles (MBGNs) exhibit excellent biocompatibility, bioactivity, osteogenic, and angiogenic properties. When interacting with polymer matrices and biomolecules, they can enhance mechanical strength and biological activity [10]. Hydrogels can mimic the structure of the extracellular matrix (ECM), providing a 3D environment for cell adhesion, growth, and proliferation. Studies have shown that the biomimetic composite hydrogel Gel-OCS/MBGN has good osteogenic and angiogenic properties. When combined with EV, Gel-OCS/MBGN can improve cell migration, proliferation, and chondrogenic differentiation, as well as promote the formation of glycosaminoglycans, extracellular matrix, and type II collagen [11].

The research by Guanghui Xu et al. showed that EVs derived from HMSC-mediated miR-21 and miR-19b could regulate the apoptosis and differentiation of neurons in spinal cord injury patients [12]. In addition, studies have shown that c-Myc transported by extracellular vesicles derived from tumors promotes the proliferation of bronchial cells in the lungs through miR-19b and miR-92a [13]. Previous studies have demonstrated that miRNAs could regulate cell apoptosis, proliferation, and differentiation processes. SMURF1 targets miR-19b-3p modified bone marrow-derived mesenchymal stem cells composite with PLLA scaffold have been proven to enhance osteogenic activity and repair critical bone defects [14]. Ao Xiong et al. found that miR-19b-3p could suppress the expression of a negative regulator of osteogenesis, SMURF1, and confirmed that introducing lentivirus pLVTHM-miR19b-3p into BMSCs could promote osteogenic differentiation of BMSCs by inhibiting the expression of SMURF1 [14]. miR-19b-3p could interact with lncRNA H19 to promote proliferation and osteogenic differentiation of bone marrow mesenchymal stem cells [15]. miR-19b-3p could also inhibit osteogenic activity and accelerate spinal cord injury-induced bone defects by regulating the PTEN/Akt/mTOR signaling pathway, indicating that miR-19b-3p is one of the potential targets for treating post-spinal cord injury bone loss [16].

miRNAs play a critical role in bone homeostasis by regulating the expression of key genes associated with osteoblast and osteoclast function [17]. miR-19b is one of the important components in the exosomes of mesenchymal stem cells [18]. During osteogenic differentiation, upregulation of miR-19b may significantly increase the transcription and translation of osteogenic factor genes [17]. In addition, miR-19b could enhance the osteogenic differentiation of mesenchymal stem cells and promote fracture healing through the KLF5/β-catenin signaling pathway mediated by WWP1/SMURF2 [19].

This study focuses on exploring the exosomes derived from bone marrow mesenchymal stem cells loaded with biomimetic composite hydrogel and promoting osteogenic differentiation by mediating the miR-19b/WWP1 axis to regulate new bone formation in rat bone defects, thereby enhancing the effect of promoting bone defect repair. This technique provides a new practical basis and theoretical foundation for treating bone defects.

## Results

### Successfully loading EVs into biomimetic composite hydrogel Gel-OCS/MBGN

**Fig. 1:**
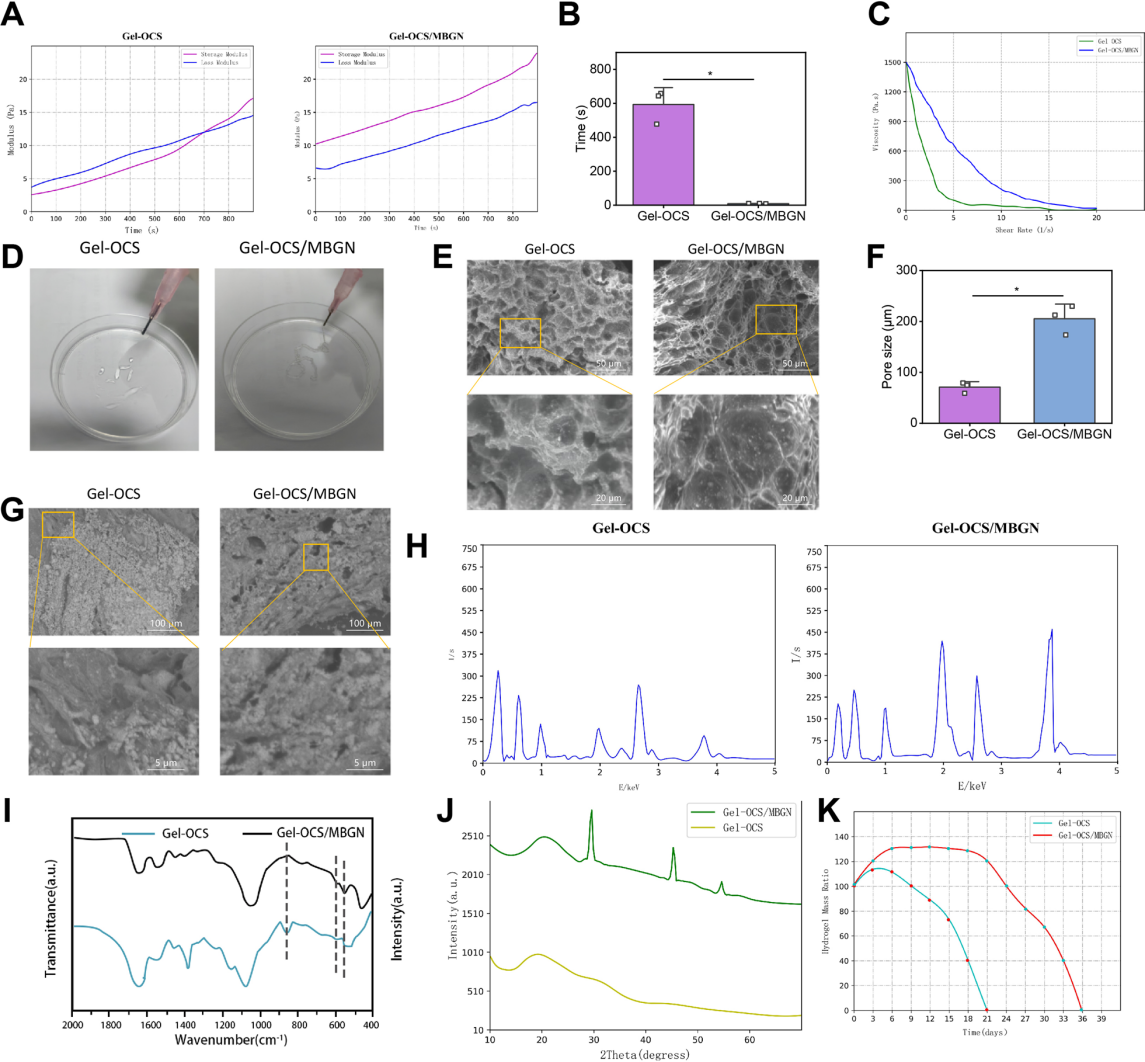
Gel-OCS/MBGN characterization. **A** Rheological analysis detected the gelation time of each group of hydrogels; **B** Rheological analysis quantified the gelation time of each group of hydrogels in a bar graph; **C** Rheological analysis detected the shear-thinning behavior of each group of Gel-OCS/MBGN hydrogels; **D** Rheological analysis detected the forming state of each group of hydrogels; **E** SEM observation detected the average pore size of each group of hydrogels, scale bar: 50 μm, scale bar: 20 μm; **F** SEM observation quantified the average pore size of each group of hydrogels in a bar graph; **G** SEM observation detected the in vitro mineralization of each group of hydrogels, scale bar: 100 μm; **H** EDS spectra observed the in vitro mineralization of each group of hydrogels; **I** FTIR spectra detected the in vitro mineralization of each group of hydrogels; **J** XRD spectra detected the in vitro mineralization of each group of hydrogels; **K** Degradation rate of each group of hydrogels

To evaluate the gelation time of Gel-OCS and Gel-OCS/MBGN hydrogels, we conducted a comprehensive rheological scanning analysis at the physiological temperature of 37 °C. We found that the Gel-OCS group hydrogel has higher storage capacity than energy loss after 640 s of reaction, and the hydrogel undergoes gelation. Compared with the Gel-OCS group, Gel-OCS/MBGN group underwent gelation after a mere 10 s of contact with water, and the gelation process was undisturbed (Fig. 1A). There is a significant difference in gelation time between the Gel-OCS/MBGN and Gel-OCS groups (p < 0.001), with the gelation time of the water gel in the Gel-OCS/MBGN group being 64 times faster than that of the Gel-OCS group (Fig. 1B). Compared with the Gel-OCS group, the Gel-OCS/MBGN group hydrogel showed better shear thinning behavior (Fig. 1C). The injection of Gel-OCS composite hydrogel resulted in an inhomogeneous shape in the syringe, while Gel-OCS/MBGN composite hydrogel showed a complete shape compared to Gel-OCS composite hydrogel (Fig. 1D). Compared with the Gel-OCS group, SEM imaging showed that Gel-OCS/MBGN hydrogel had a larger pore size without damaging the porous structure of hydrogel, which is beneficial for bone tissue growth (Fig. 1E, F, p < 0.001). After soaking in simulated body fluid (SBF) for 7 days, cauliflower-shaped HA crystals were found on the surface of Gel-OCS/MBGN hydrogel by SEM imaging (Fig. 1G). Compared with the Gel-OCS group, the intensity of Ca and P peaks in the EDS spectrum Gel-OCS/MBGN group hydrogels was higher (Fig. 1H). Compared with the Gel-OCS group, the FTIR spectrum showed that in the Gel-OCS/MBGN group, two characteristic bands appeared at ~ 560 and ~ 600 cm^−1^, indicating the formation of crystalline HA phase in the hydrogel, with stronger band intensity (Fig. 1I). XRD analysis confirmed that after soaking in SBF for 7 days, crystalline HA was formed on the surface of the Gel-OCS/MBGN composite hydrogel (Fig. 1J). After 21 days in PBS (pH = 7.4), Gel-OCS group hydrogel completely degraded, while Gel-OCS/MBGN group hydrogel degraded completely after 36 days in PBS, demonstrating a slower degradation rate (Fig. 1K).

The above results have shown that Gel-OCS/MBGN hydrogel has good biological properties and could be applied in bone regeneration and repair [10]. Past literature research has shown that EVs from BMSCs could promote bone defect repair [20, 21]. However, direct injection of EVs into the bone defect site leads to a significant decrease in performance due to the fluidity of the liquid, which causes the EVs to disperse in the body. Therefore, we loaded EVs into Gel-OCS/MBGN hydrogels. The purpose of doing so is to fix the EVs in the bone defect area on the one hand and, on the other hand, to enhance the promoting repair effect through slow release.

We first demonstrated the adipogenic, osteogenic, and chondrogenic differentiation abilities of BMSCs through induction (Additional file 1: Fig. S1A-B). Flow cytometry analysis showed that CD90 and CD44 were positively expressed in BMSCs, while IgG, CD45, and CD34 were negatively expressed (Additional file 1: Fig. S1C). Subsequently, we obtained BMSCs EVs using the differential centrifugation method. TEM observation revealed that BMSCs-EVs exhibited a round or elliptical shape with membranous vesicular structures (Additional file 1: Fig. S1D). NTA detection revealed that the particle size of EVs is around 120 nm (Additional file 1: Fig. S1E). Western blot was used to detect the expression of EV markers, and the results showed high expression of CD9, HSP70, and CD81, which are specific surface markers of EVs, but no expression of endoplasmic reticulum marker calnexin (Additional file 1: Fig. S1F), confirming the successful isolation of EVs.

**Fig. 2:**
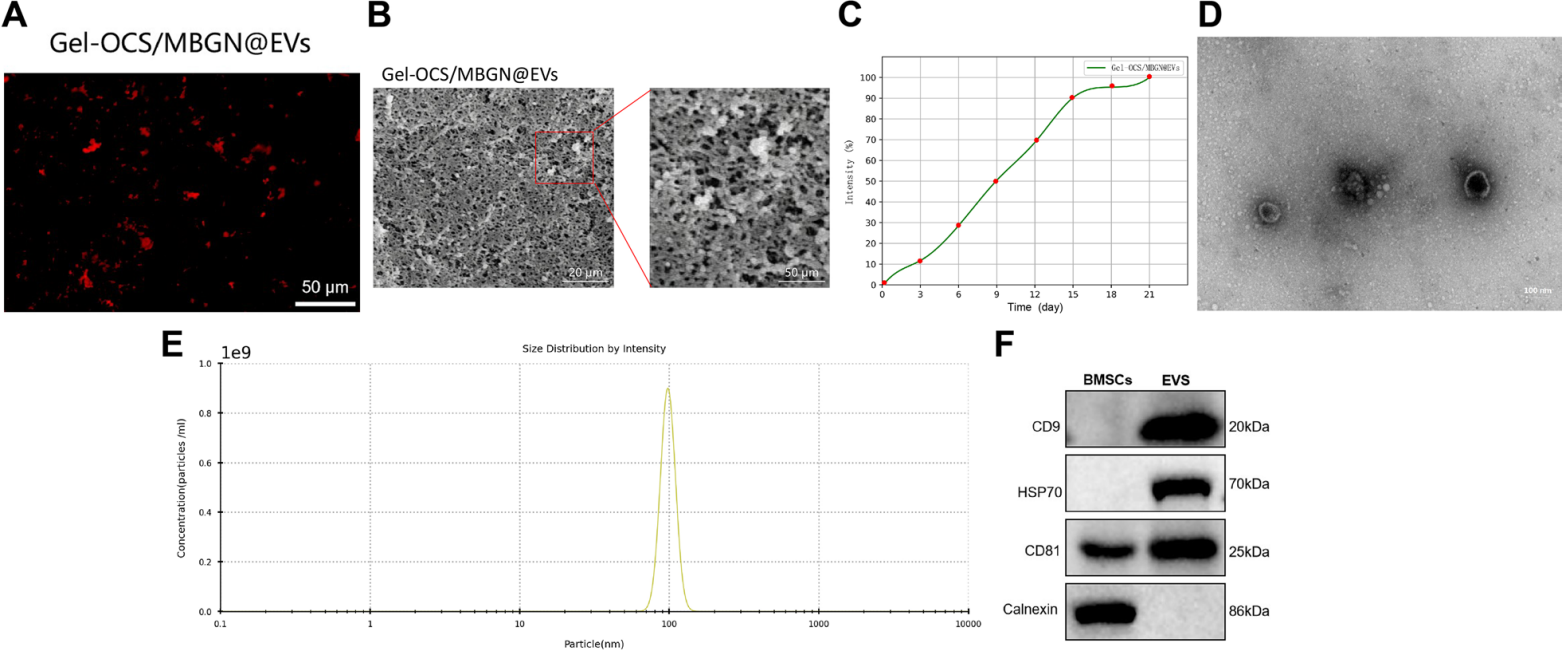
Characterization of Gel-OCS/MBGN@EVs. *Note* **A** Fluorescence microscopy was used to observe whether EVs were successfully loaded into Gel-OCS/MBGN. DiR-labeled EVs appear red, scale bar: 50 μm; **B** SEM was used to observe the morphology of Gel-OCS/MBGN@EVs, The left scale is 20 μm, and the right scale is 50 μm; **C** The release curve of EVs in Gel-OCS/MBGN@EVs; **D** TEM was used to observe the morphology of released EVs from Gel-OCS/MBGN@EVs, scale bar: 100 μm; **E** NTA was used to detect the particle size of EVs released from Gel-OCS/MBGN@EVs; **F** Western blot was used to detect the expression of marker proteins in EVs released from Gel-OCS/MBGN@EVs. The experiment was repeated three times, and "ns" indicates no significant difference between the two groups

Subsequently, mix the DiR-labeled EVs with Gel-OCS/MBGN solution. In the inverted fluorescence microscope (Fig. 1A), abundant red fluorescent EVs could be observed, which are absorbed by Gel-OCS/MBGN, a red fluorescent dye. Spherical microvesicles with diameters of approximately 50–150 nm, which are EVs, could be observed adhering to the Gel-OCS/MBGN surface in SEM imaging (Fig. 2B, C). This result proves that EVs have been successfully loaded into Gel-OCS/MBGN. We further tested whether Gel-OCS/MBGN@EVs could effectively release EVs. We detected the release and labeled DiR EVs using the dialysis method. As shown in Fig. 2C, the release concentration of EVs gradually increased over time. Within 21 h, the release of EVs could reach 95%. The release of EVs is highly correlated with time, indicating that Gel-OCS/MBGN hydrogels have good sustained release activity for EVs. In addition, we performed activity assays on the dialyzed EVs. TEM (Fig. 2D) reveals typical circular nanoparticles with a size range of approximately 50–150 nm. NTA (Fig. 2E) showed a similar size distribution. The Western blot (Fig. 2F) detection results showed significant expression of surface markers CD9, HSP70, and CD81 in EVs, while the calcium-binding protein Calnexin was not expressed. It indicates that Gel-OCS/MBGN hydrogel could effectively maintain the activity of EVs.

### Gel-OCS/MBGN@EVs exhibit good cellular compatibility

**Fig. 3:**
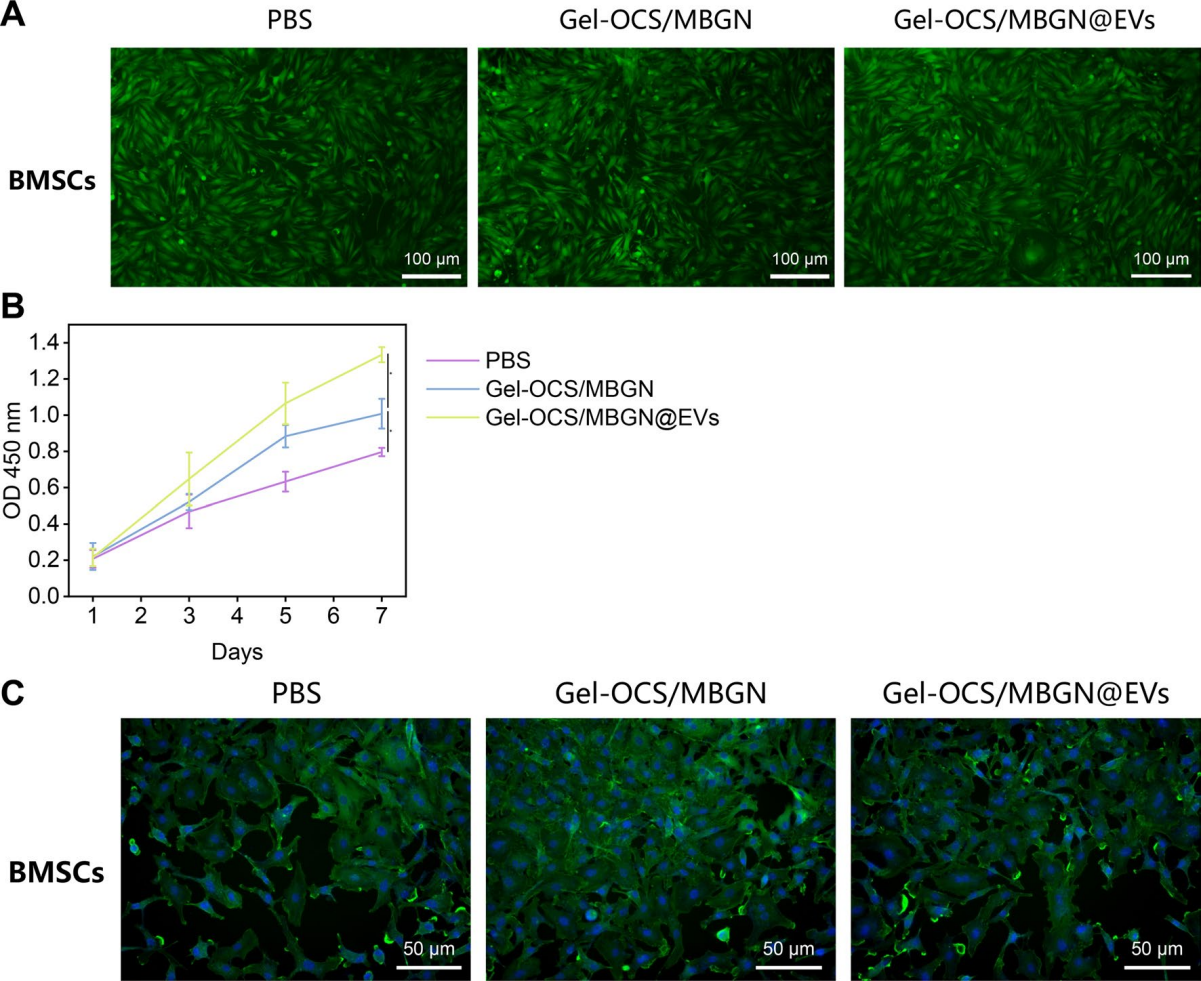
Toxic effect of Gel-OCS/MBGN@EVs on cells. *Note* **A** Live/dead staining was performed to assess the viability of BMSCs in each group. Green cells indicate live cells, while red cells indicate dead cells. Scale bar: 100 μm; **B** CCK-8 assay was used to evaluate the survival and proliferation of BMSCs in each group; **C** Immunofluorescence staining was conducted to examine the adhesion of BMSCs in each group. Phalloidin was used to stain the cytoskeleton of cells in green, while DAPI was used to stain the nuclei in blue. Scale bar: 50 μm. * indicates that the comparison between the two groups is statistically significant with P < 0.05, and the cell experiment is repeated three times

To investigate the biocompatibility of Gel-OCS/MBGN@EVs, we examined their in vitro cytotoxicity. Firstly, we incubated the BMSCs with Gel-OCS/MBGN and Gel-OCS/MBGN@EVs to observe the cell vitality, with the PBS group as the control. High viability of BMSCs was observed on all groups of hydrogels through live/dead staining, and almost no dead cells were detected (Fig. 3A, B). Further, evaluate the proliferation of BMSCs using CCK-8 assay. After incubating three types of cells separately in hydrogels, the detection was performed on days 1, 3, 5, and 7 of incubation. As shown in Fig. 3B, the OD values of cells in each group increased with time. Starting from day 3, the OD values of cells in the hydrogel group increased significantly, with the Gel-OCS/MBGN@EVs group showing the highest increase compared to the PBS group, followed by the Gel-OCS/MBGN group. It indicates that hydrogels are non-toxic to BMSCs and could promote their proliferation. To further validate the effects of Gel-OCS/MBGN@EVs on cell adhesion and spreading, after incubation for 24 h, cell skeletons and nuclei were stained and observed under a fluorescence microscope. The results showed that cells spread well on the surface of Gel-OCS/MBGN hydrogels and Gel-OCS/MBGN@EVs hydrogels (Fig. 3C).

In summary, the above results indicate that Gel-OCS/MBGN@EVs have high cell compatibility, no significant toxic effects, and could promote cell proliferation and diffusion.

### Gel-OCS/MBGN@EVs could promote the osteogenic differentiation of BMSCs

**Fig. 4:**
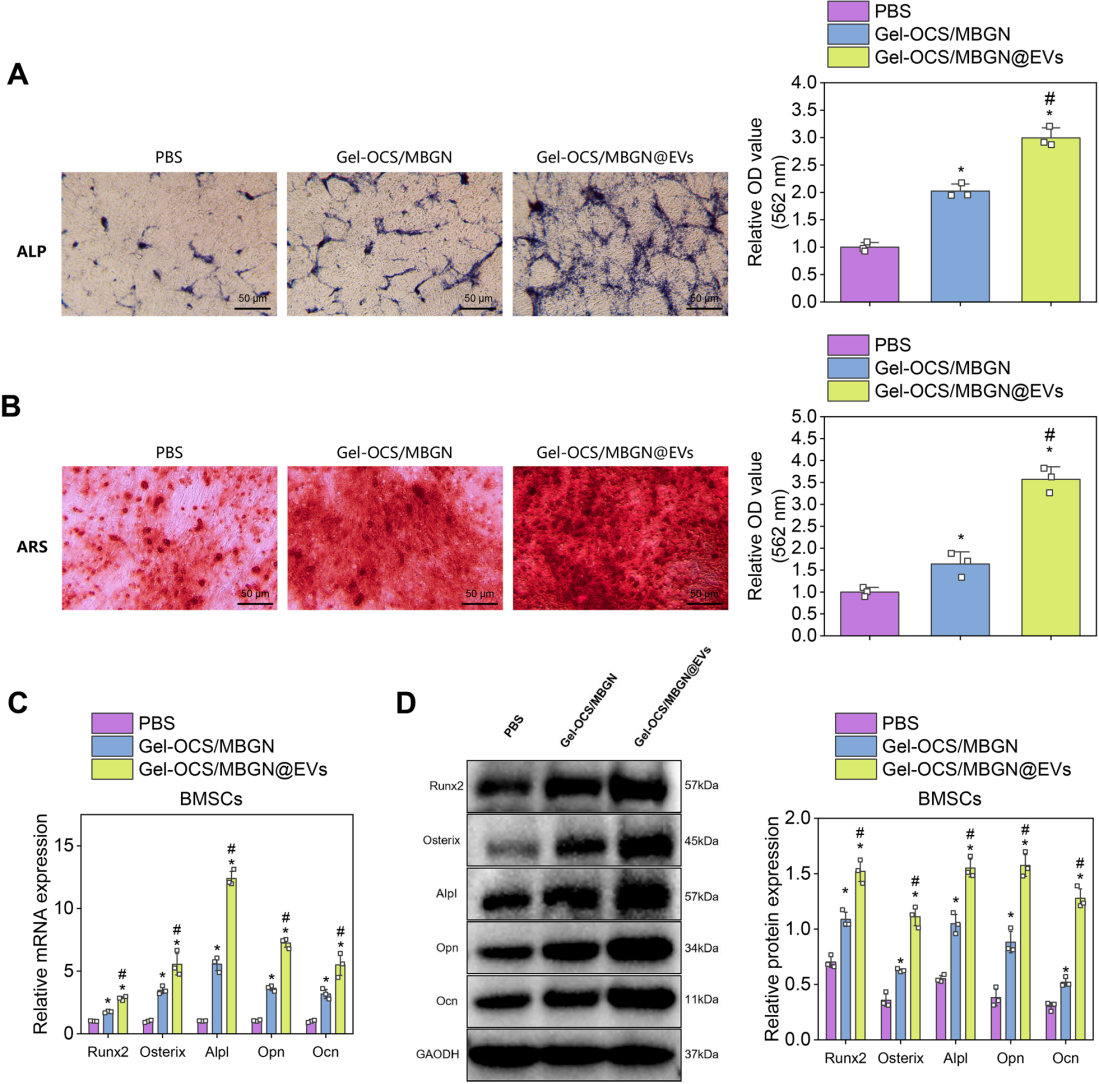
The effect of Gel-OCS/MBGN@EVs on the osteogenic differentiation of BMSCs. *Note* **A** ALP activity of BMSCs in each group was detected by ALP staining, scale bar: 50 μm; **B** Osteogenic differentiation of BMSCs in each group was detected by ARS staining, scale bar: 50 μm; **C**, **D** The gene and protein expression levels of osteogenic markers Runx2, Osterix, Alpl, Opn, and Ocn in BMSCs of each group were detected by RT-qPCR and Western blot, respectively (* indicates P < 0.05 compared with the PBS group, # indicates P < 0.05 compared with the Gel-OCS/MBGN group, and the cell experiments were repeated three times)

To investigate the effect of Gel-OCS/MBGN-loaded EVs on the osteogenic differentiation of BMSCs, we cultured BMSCs in different groups of hydrogels. We observed the effects of each treatment on the osteogenic differentiation of BMSCs. First, ALP and ARS staining was performed on the 7th and 21st days of inducing BMSCs osteogenic differentiation. The results showed that compared with the PBS group, ALP and ARS staining were significantly intensified in the Gel-OCS/MBGN@EVs and Gel-OCS/MBGN groups, indicating that Gel-OCS/MBGN@EVs and Gel-OCS/MBGN significantly promoted the osteogenic differentiation of BMSCs. Compared with the Gel-OCS/MBGN group, the osteogenic differentiation of BMSCs was significantly enhanced in the Gel-OCS/MBGN@EVs group (Fig. 4A, B).

Subsequently, expression of osteogenic differentiation markers Runx2, Osterix, Alpl, Opn, and Ocn in BMSCs was detected via RT-qPCR and Western blot. The results showed that compared with the PBS group, the expression levels of osteogenic differentiation markers of BMSCs in Gel-OCS/MBGN@EVs and Gel-OCS/MBGN groups were significantly increased. Compared with the Gel-OCS/MBGN group, osteogenic differentiation markers' expression levels in BMSCs were significantly increased in the Gel-OCS/MBGN@EVs group (Fig. 4C, D). The above results indicate that Gel-OCS/MBGN-loaded EVs significantly promote osteogenic differentiation of BMSCs.

### The screening results of bioinformatics suggest that WWP1 may be a key gene promoting the osteogenic differentiation of BMSCs

**Fig. 5:**
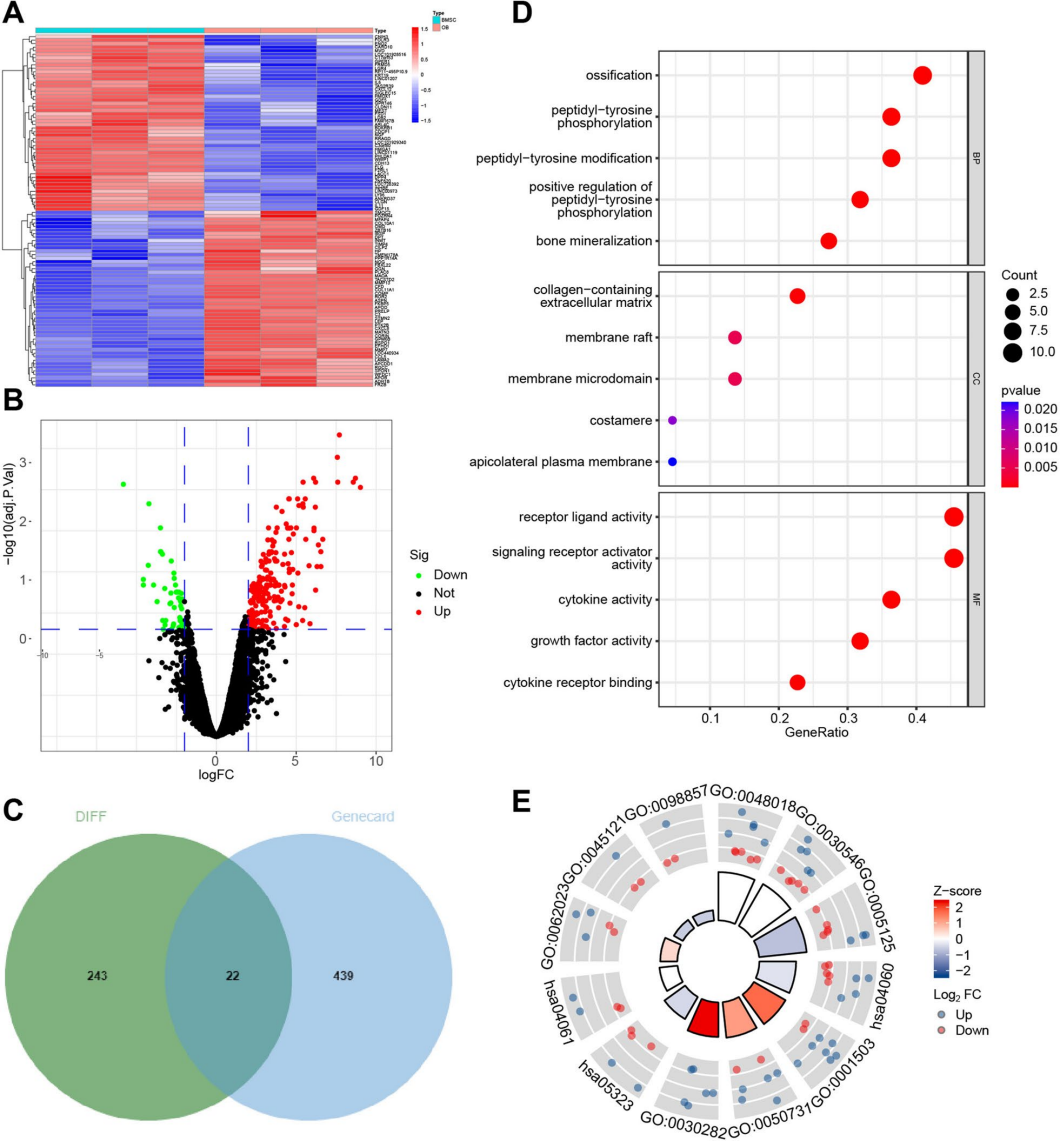
Candidate gene screening for osteogenic differentiation of BMSCs. *Note* **A** Heatmap of differential analysis of high-throughput sequencing data (BMSC group, n = 3; osteoblast (OB) group, n = 3); **B** Volcano plot of differential analysis of high-throughput sequencing data. Red dots represent up-regulated genes, green dots represent down-regulated genes, and black dots represent genes with no significant differential expression (BMSC group, n = 3; osteoblast (OB) group, n = 3); **C** Venn diagram of the intersection between Genecard database and differentially expressed genes obtained by high-throughput sequencing data analysis; **D** GO enrichment analysis results of candidate genes, and the horizontal axis represents GeneRatio; **E** Circos plot of KEGG-GO integrated analysis of candidate genes

**Fig. 6:**
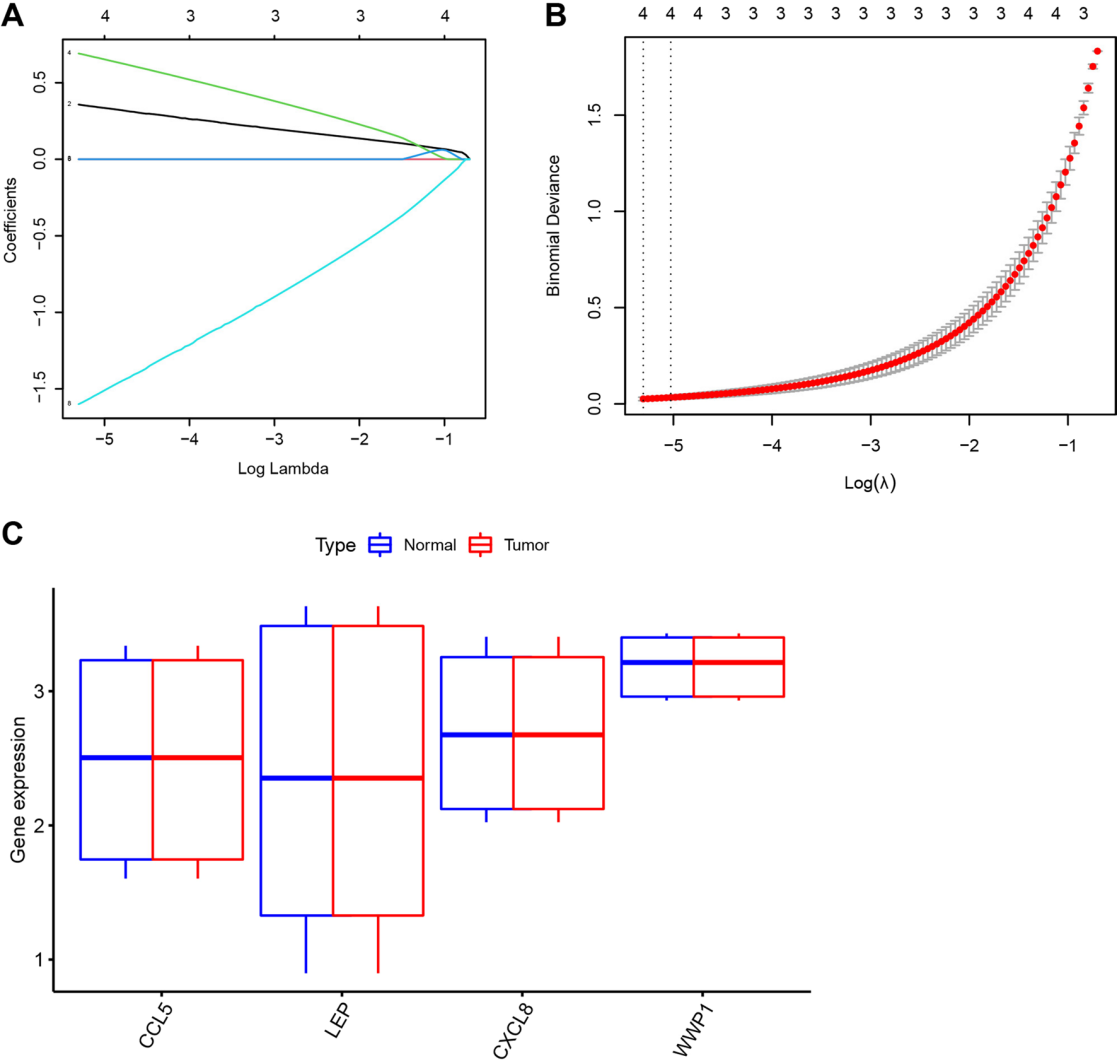
Key gene selection for osteogenic differentiation of BMSCs. *Note* **A** Lasso coefficient path diagram of differentially expressed genes in RNA-seq; **B** Cross-validation curve of differentially expressed genes in RNA-seq; **C** Expression levels of CCL5, LEP, CXCL8, and WWP1 in high-throughput sequencing data of BMSC group (n = 3) and osteoblast (OB) group (n = 3)

By high-throughput sequencing, expression profile data related to osteogenesis differentiation of rat BMSCs were obtained, with |log2FC| > 2 and adj.P < 0.05 as the threshold, 265 genes (215 up-regulated and 50 down-regulated) significantly differentially expressed in the osteogenesis differentiation of BMSCs were screened (Fig. 5A, B). Using the Genecard database to retrieve genes related to "BMSCs osteogenic differentiation", 461 results were obtained. By taking the intersection through Venn diagrams, a total of 22 overlapping target genes were obtained (Fig. 5C). GO and KEGG enrichment analysis of candidate target genes revealed their involvement in biological processes such as ossification, positive regulation of peptidyl-tyrosine phosphorylation, bone mineralization, cytokine-cytokine receptor interaction, and rheumatoid arthritis (Fig. 5D, E, Additional file 2: Table S1). Further, obtain key factors involved in osteogenic differentiation through machine learning algorithms. A model was constructed by performing Lasso regression on the 22 differentially expressed genes obtained above, and 4 core genes were identified: CCL5, LEP, CXCL8, and WWP1 (Fig. 6A, B). Among these four genes, only WWP1 was significantly down-regulated in osteoblasts, while CCL5, LEP, and CXCL8 expression was significantly up-regulated in osteoblasts (Fig. 6C). WWP1 is considered an inhibitor of osteoblast differentiation [22]. Therefore, we will select WWP1 for further study, including its upstream miRNA and related mechanisms.

### EVs may inhibit the expression of WWP1 by delivering miR-19b-3p

**Fig. 7:**
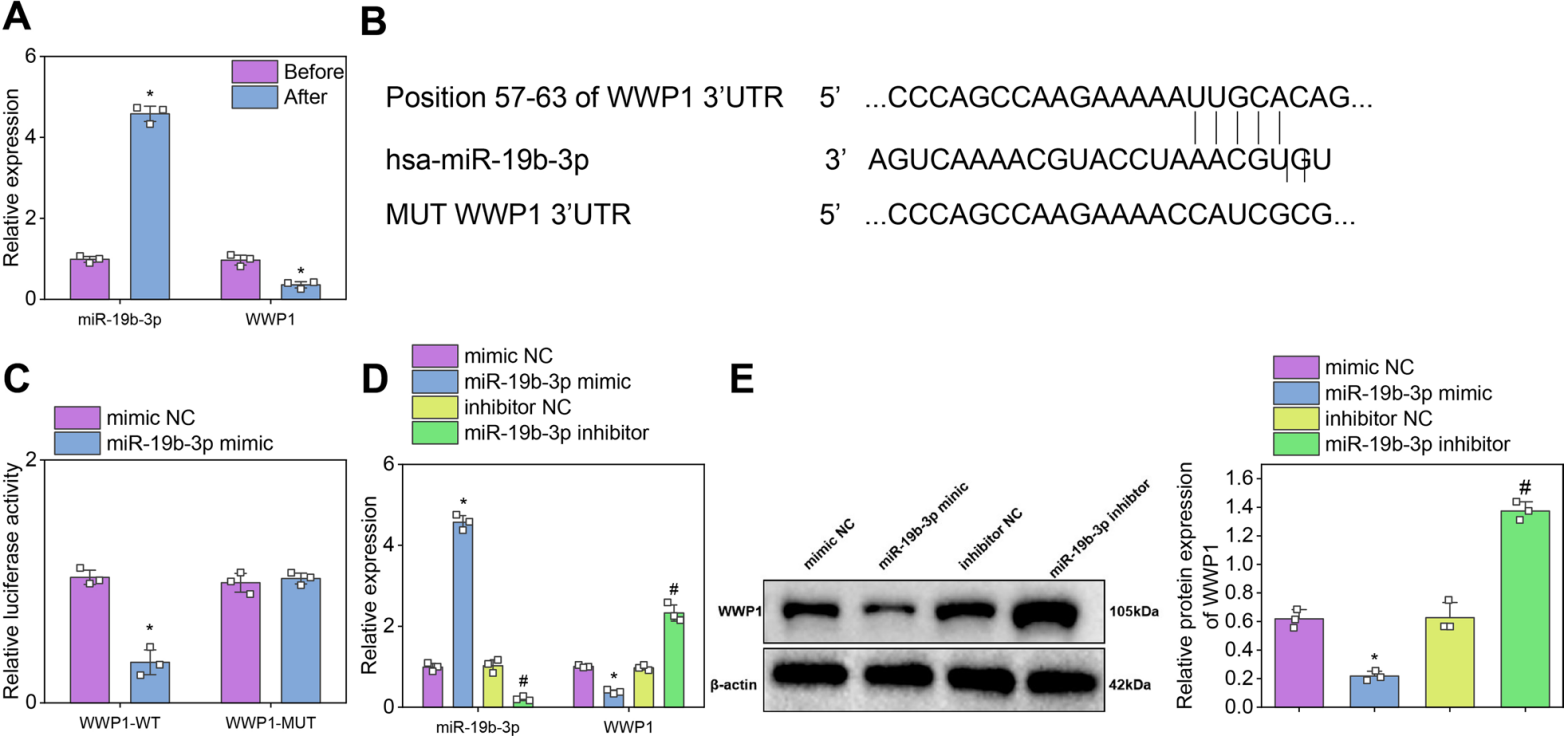
Regulatory role of miR-19b-3p on WWP1. *Note* **A** Expression levels of miR-19b-3p and WWP1 in the osteogenic differentiation of BMSCs were detected by RT-qPCR (n = 12 per group, * indicates significantly different from the Sham group with P < 0.05). **B** TargetScan was used to predict the binding sites of miR-19b-3p and WWP1. **C** A dual luciferase reporter assay was performed to confirm the targeting relationship between miR-19b-3p and WWP1 (experiments were repeated three times, * indicates a significantly different from the mimic NC group with P < 0.05). **D** RT-qPCR detected expression levels of miR-19b-3p and WWP1 in cells from each group (experiments were repeated three times, * indicates significantly different from the mimic NC group with P < 0.05, # indicates significantly different from the inhibitor NC group with P < 0.05). **E** The protein expression levels of WWP1 in cells from each group were detected by Western blot (experiments were repeated three times, * indicates significantly different from the mimic NC group with P < 0.05, # indicates significantly different from the inhibitor NC group with P < 0.05)

RT-qPCR analysis showed that the expression of miR-19b-3p significantly increased while that of WWP1 significantly decreased in BMSCs undergoing osteogenic differentiation (Fig. 7A).

To investigate whether miR-19b-3p could target WWP1, the binding site between miR-19b-3p and WWP1 was obtained from the TargetScan database (Fig. 7B). Subsequently, the results of the dual-luciferase reporter gene assay showed that in WWP1-WT, compared with the mimic NC group, there was a significant decrease in luciferase activity in BMSCs cells of the miR-19b-3p mimic group (Fig. 7C), indicating that miR-19b-3p could target and inhibit WWP1.

Further research indicates that miR-19b-3p could regulate the expression of WWP1. The results of RT-qPCR and Western blot detection showed that in the miR-19b-3p mimic group, the expression level of miR-19b-3p in BMSCs cells was significantly increased, while the expression level of WWP1 was significantly decreased; In the miR-19b-3p inhibitor group, the expression level of miR-19b-3p in the cells was significantly decreased, while the expression level of WWP1 was significantly increased (Fig. 7D, E).

In conclusion, miR-19b-3p could regulate osteogenic differentiation by targeting and inhibiting the expression of WWP1.

### EVs could transfer miR-19b-3p to suppress WWP1 expression and promote osteogenic differentiation

**Fig. 8:**
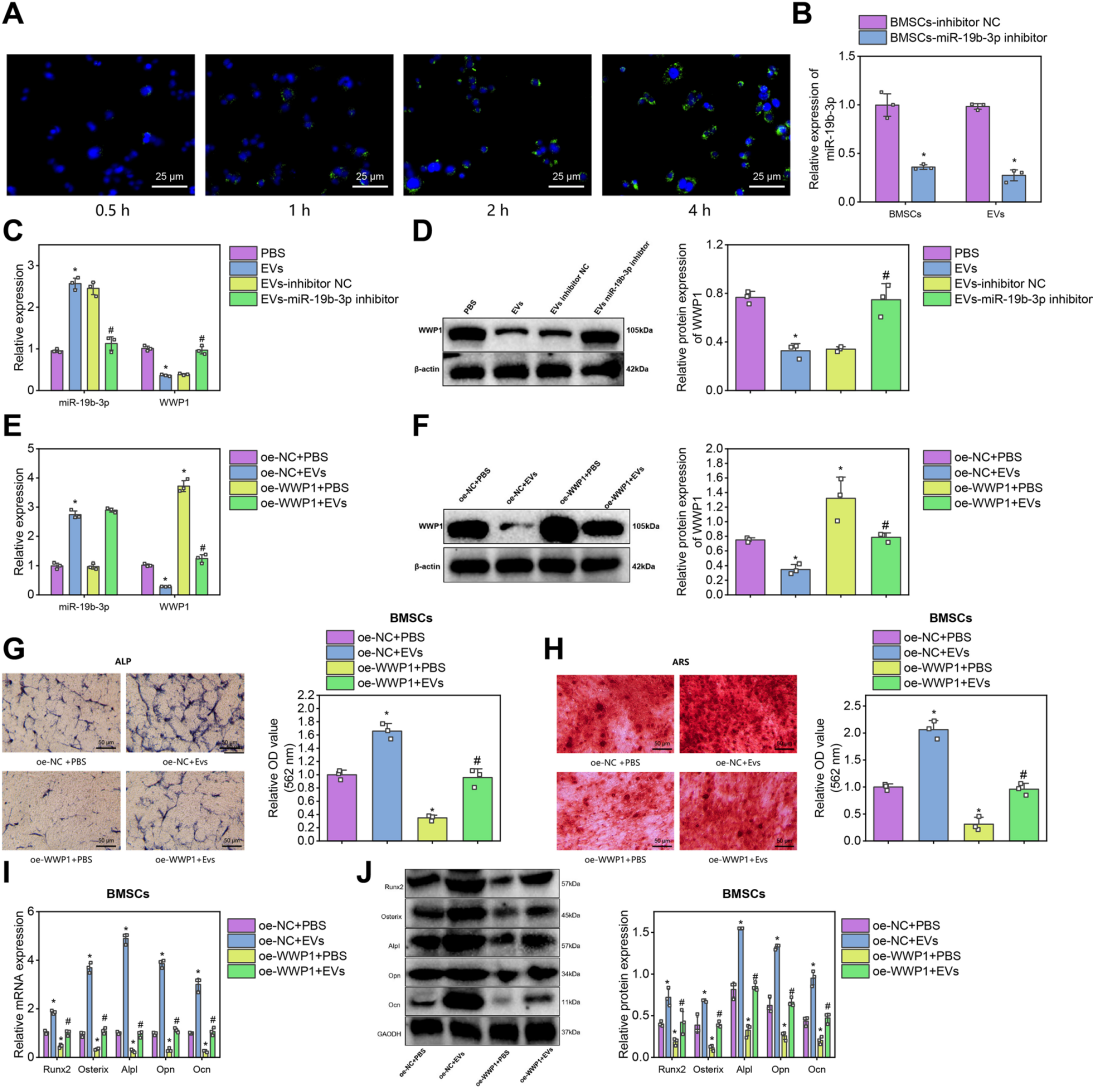
Regulatory effects of miR-19b-3p delivered by EVs derived from BMSCs on the WWP1 axis. *Note* **A** BMSCs uptake of EVs at different time points was observed by confocal fluorescence microscopy, scale bar: 25 μm; **B** The expression of miR-19b-3p in BMSCs and BMSC-derived EVs was detected by RT-qPCR (n = 12 per group, * indicates P < 0.05 compared to the BMSCs-inhibitor NC group); **C** The gene expression levels of miR-19b-3p and WWP1 were detected by RT-qPCR in various groups of cells (experiment repeated three times, * indicates P < 0.05 compared with the PBS group, # indicates P < 0.05 compared with the EVs-inhibitor NC group); **D** The protein expression level of WWP1 in each group of cells was detected by Western blot (experiment repeated three times, * indicates P < 0.05 compared with the PBS group, # indicates P < 0.05 compared with the EVs-inhibitor NC group); **E** The gene expression levels of miR-19b-3p and WWP1 were detected by RT-qPCR in various groups of cells (experiment repeated three times, * indicates P < 0.05 compared to the oe-NC + PBS group, # indicates P < 0.05 compared to the oe-NC + EVs group); **F** The protein expression level of WWP1 in each group of cells was detected by Western blot (experiment repeated three times, * indicates P < 0.05 compared to the oe-NC + PBS group, # indicates P < 0.05 compared to the oe-NC + EVs group); **G** ALP staining was used to detect the activity of ALP in various groups of BMSCs, scale bar: 50 μm; **H** ARS was used to detect osteogenic differentiation of BMSCs in each group, scale bar: 50 μm (experiment repeated three times, * indicates P < 0.05 compared to the oe-NC + PBS group, # indicates P < 0.05 compared to the oe-NC + EVs group); **I**, **J** RT-qPCR and Western blot were used to detect the gene and protein expression levels of osteogenic differentiation markers Runx2, Osterix, Alpl, Opn, and Ocn in various groups of BMSCs. The experiment was repeated three times. * indicates P < 0.05 compared with the oe-NC + PBS group, # indicates P < 0.05 compared with the oe-NC + EVs group

We first test whether EVs could transmit miR-19b-3p and regulate the expression of WWP1. We labeled BMSCs-EVs with DiO fluorescence and co-cultured them with BMSCs for 24 h. We used confocal fluorescent microscopy to observe, and the results showed that with the prolonged incubation time, many EVs entered the cytoplasm of BMSCs (Fig. 8A). We also used qRT-PCR to detect the silencing effect of miR-19b-3p in BMSCs and detected the expression of miR-19b-3p in both BMSCs and their derived EVs. The results showed that after silencing miR-19b-3p in BMSCs, the expression levels of miR-19b-3p in BMSCs and EVs were significantly decreased. Compared with the BMSCs-inhibitor NC group, the expression level of miR-19b-3p was significantly decreased in the BMSCs-miR-19b-3p inhibitor group (Fig. 8B).

Next, we will co-culture BMSCs with or without EVs and incubate EVs extracted from transfected BMSCs with BMSCs cells. We used qRT-PCR and Western blot to detect that the expression level of miR-19b-3p in EVs derived from BMSCs significantly increased after silencing miR-19b-3p, while the expression level of WWP1 significantly decreased. Compared with the PBS group, the expression level of miR-19b-3p in BMSCs was significantly increased, and the expression level of WWP1 was significantly decreased in the EVs group. Compared with the EVs-inhibitor NC group, the expression level of miR-19b-3p in BMSCs was significantly reduced in the EVs-miR-19b-3p inhibitor group, while the expression level of WWP1 was significantly increased (Fig. 8C, D). The above results indicate that EVs derived from BMSCs could target and inhibit the expression of WWP1 by delivering miR-19b-3p into BMSCs.

Then, we further investigated the impact of EVs derived from BMSCs on cell functions by delivering miR-19b-3p to suppress the expression of WWP1. We detected the expression of the osteogenic differentiation markers Run 2, Osterix, Alpl, Opn, and Ocn in BMSCs by qRT-PCR and Western blot. Results showed that compared with the oe-NC + PBS group, the expression of bone differentiation markers significantly increased in the oe-NC + EVs group, decreased significantly in the oe-WWP1 + PBS group, and decreased significantly in the oe-WWP1 + EVs group compared with the oe-NC + EVs group. Meanwhile, the result was confirmed using ALP and ARS staining (Fig. 8E–J).

Therefore, our study demonstrates that EVs derived from BMSCs could promote osteogenic differentiation of BMSCs by regulating WWP1 expression through transferring miR-19b-3p.

### Biomimetic composite hydrogel Gel-OCS/MBGN promotes bone defect repair through regulation of the miR-19b-3p/WWP1 axis by loading EVs

**Fig. 9:**
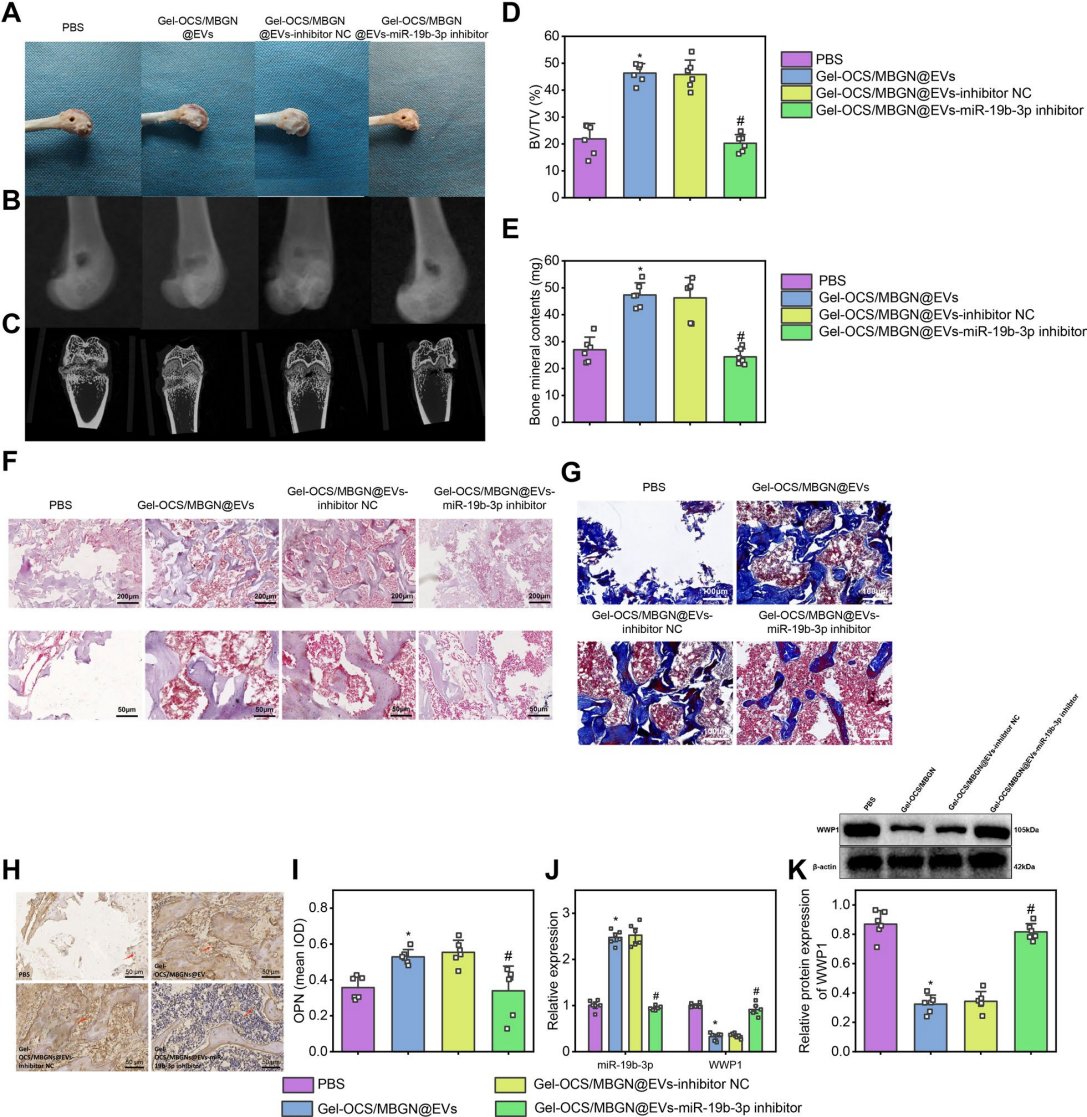
Bionic composite hydrogel Gel-OCS/MBGN regulates bone defect healing through loading EVs to modulate the miR-19b-3p/WWP1 axis. *Note* **A** Observing the healing of femurs in each group of rats by naked eye; **B**, **C** Observing the new bone formation at the site of femoral defect in each group of rats by X-ray and Micro-CT; **D**, **E** BV/TV and BMC analysis in (**C**) (* indicates P < 0.05 compared with PBS group, # indicates P < 0.05 compared with Gel-OCS/MBGN@EVs-inhibitor NC group); **F** Pathological changes in new bone formation at the site of femoral defect in each group of rats detected by H&E staining, Scale bar: 200 μm; **G** Collagen fiber formation in new bone formation at the site of femoral defect in each group of rats detected by Masson staining, Scale bar: 100 μm; **H**, **I** Expression of OPN, a marker of osteogenic differentiation, detected by immunohistochemical staining in each group of rats at the site of femoral defect (cytoplasm), Scale bar: 50 μm, red arrows indicate OPN-positive cells; **J** Expression levels of miR-19b-3p and WWP1 at the site of femoral defect in each group of rats detected by RT-qPCR (N = 6, * indicates P < 0.05 compared with PBS group, # indicates P < 0.05 compared with Gel-OCS/MBGN@EVs-inhibitor NC group). **K** Western blot was used to detect the protein expression levels of WWP1 in the femoral defect of each group (N = 6, * indicates P < 0.05 compared with the PBS group, # indicates P < 0.05 compared with the Gel-OCS/MBGN@EVs-inhibitor NC group)

We set up a total of four experimental groups: the PBS (blank control) group, Gel-OCS/MBGN group, Gel-OCS/MBGN@EVs-inhibitor NC group, and Gel-OCS/MBGN@EVs-miR-19b-3p inhibitor group. We retrieved the femurs for further investigation at the 8th-week post-implantation of hydrogel in a rat model. According to the results shown in Fig. 9A, Gel-OCS/MBGN group covered the defect area with newly regenerated tissue compared to the PBS group. The junction of the defect showed almost completely matured bone tissue with complete and continuous bone closure. Gel-OCS/MBGN@EVs-inhibitor NC group showed complete and smooth regeneration of new tissue, which was well integrated with the surrounding primitive cartilage, forming a morphology similar to that of normal cartilage. Compared with the Gel-OCS/MBGN@EVs-inhibitor NC group, a small amount of regenerative new bone tissue and less bone formation were observed on the bone surface in the Gel-OCS/MBGN@EVs-miR-19b-3p inhibitor group. The surface of the newly-formed tissue was rough, and there was no complete and continuous bone closure, which could not be combined with cartilage.

Meanwhile, the X-ray results showed that the bone defects in the PBS group remained relatively large, while in comparison, the bone pores in the Gel-OCS/MBGN rats were significantly reduced, and their bone defects had almost fully healed. Additionally, a remodeling process was observed, indicating accelerated bone repair. Compared with the Gel-OCS/MBGN@EVs-inhibitor NC group, the healing of bone defects in rats was reduced, the speed of reshaping and repairing was decreased, the bone porosity in rats was increased, and a small amount of new autologous bone tissue was formed around the bone defect site in the Gel-OCS/MBGN@EVs-miR-19b-3p inhibitor group (Fig. 9B).

Through Micro-CT imaging, we observed the formation of new bone tissue at the bone defect site in rats. We found that only a small amount of new bone was formed in the PBS group rats at the defect site. In comparison, the Gel-OCS/MBGN group rats had increased bone volume, number of trabeculae, and bone formation rate, indicating a significant increase in new bone formation. Compared with Gel-OCS/MBGN@EVs-inhibitor NC, the Gel-OCS/MBGN@EVs-miR-19b-3p inhibitor group showed limited new bone formation. The number of trabeculae and the bone formation rate increase or decrease, and the bone structure and density increase or decrease (Fig. 9C).

Quantitative analyses of bone volume fraction (BV/TV) and bone mineral content (BMC) showed that compared with the PBS group, BV/TV and BMC were significantly increased in the Gel-OCS/MBGN group, indicating a significant increase in bone quantity and mineral content. Compared with the Gel-OCS/MBGN@EVs-inhibitor NC group, BV/TV and BMC were significantly decreased in the Gel-OCS/MBGN@EVs-miR-19b-3p inhibitor group, indicating a decrease in bone regeneration ability and a significant reduction in bone trabeculae, bone quantity, and mineral content, as well as a decrease in the ability to heal bone defects (Fig. 9D–G).

To confirm the role of Gel-OCS/MBGN@EVs in regulating osteogenic differentiation, we used immunohistochemistry to detect the expression of markers. Results showed that compared with the PBS group, there was a significant increase in the expression of OPN, a bone differentiation marker in Gel-OCS/MBGN. Moreover, compared with Gel-OCS/MBGN@EVs-inhibitor NC group, OPN expression at the boundaries between fibrous tissues and bone tissues was significantly reduced in Gel-OCS/MBGN@EVs-miR-19b-3p inhibitor group (Fig. 9H-I). Further RT-qPCR detection revealed that compared to the PBS group, the expression level of miR-19b-3p was significantly increased, while the expression level of WWP1 was significantly decreased in the BMSCs of the Gel-OCS/MBGN group. Compared with Gel-OCS/MBGN@EVs-inhibitor NC group, the expression level of miR-19b-3p in BMSCs was significantly decreased, while the expression level of WWP1 was significantly increased in Gel-OCS/MBGN@EVs-miR-19b-3p inhibitor group (Fig. 9J). By Western blotting to detect the expression of WWP1, we found that compared with the PBS group, the WWP1 expression was significantly decreased in the Gel-OCS/MBGN group, while compared with the Gel-OCS/MBGN@EVs-inhibitor NC group, the WWP1 expression was significantly increased in the Gel-OCS/MBGN@EVs-miR-19b-3p inhibitor group (Fig. 9K, M).

Our research findings suggest that Gel-OCS/MBGN@EVs may promote bone regeneration in femoral defect rats by inhibiting WWP1 expression via delivering miR-19b-3p to facilitate osteogenic differentiation.

## Discussion

We successfully constructed a composite hydrogel GEL-OCS/MBGN and loaded EVs into it. Previous research reports have shown that using compound hydrogels as carriers could enhance EVs' stability and protective effect [23]. Moreover, combining nanomaterials with EVs could enhance the drug-delivery ability of EVs [24]. Using OCS and MBGN as materials could enhance the biocompatibility and biodegradability of the materials [25].

Mesenchymal stem cells (hBMSCs) and extracellular vesicles (EVs) in the bone marrow are potentially valuable in promoting bone regeneration. In these studies, EVs from different sources were used to promote the osteogenic differentiation of hBMSCs. For instance, EVs derived from skeletal muscle cells could promote osteogenic differentiation of hBMSCs by activating specific signaling pathways [26]. Another study showed that loading EVs derived from dental pulp stem cells into hydroxyapatite/polylactide composite materials could promote bone regeneration [27]. These research results are consistent with the findings of our study, which indicate that loading EVs into biomimetic composite hydrogel GEL-OCS/MBGN could promote rat bone defect repair by regulating the miR-19b/WWP1 axis. This result suggests that combining biologically active EVs with biocompatible carrier materials could synergistically promote bone regeneration. In addition, research on combining GEL-OCS/MBGN material with hydroxyapatite has also demonstrated that this composite material has higher biological activity and biocompatibility, further enhancing its potential applications in bone regeneration [10]. These studies provide valuable insights for future clinical applications in bone defect repair and skeletal regeneration.

During the process of osteogenesis, bone marrow mesenchymal stem cells (BMSCs) gradually differentiate into osteoblasts and secrete collagen fibers around the osteoblasts. This promotes the deposition of calcium on the collagen fibers, resulting in further transformation into osteocytes [28]. Studies have shown that miR-19b-3p enhances osteogenic activity and its high expression is associated with osteogenic differentiation [14]. Additionally, literature reports indicate that miR-19b-3p promotes osteogenic differentiation by suppressing the expression of WWP1 [19].

We identified WWP1 as a key factor in osteogenic differentiation through screening with bioinformatics and found that it is regulated by miR-19b. In vitro cell experiments demonstrated that EVs could promote osteogenic differentiation by transmitting miR-19b to suppress WWP1 expression. The results indicate that WWP1 is a key factor in osteogenic differentiation, and miR-19b regulates osteogenic differentiation by controlling the expression of WWP1. In addition, EVs could promote osteogenic differentiation by suppressing WWP1 expression through transmitting miR-19b. This study provides new clues for a deeper understanding of the molecular mechanism of bone differentiation. It provides a basis for the potential use of EVs to treat bone-related diseases.

The research results indicate that miR-19b plays a crucial role in osteogenic differentiation, promoting it by targeting WWP1 and suppressing its expression [29]. The association with the Wnt/β-catenin signaling pathway further revealed the importance of miR-19b in bone formation [19]. In addition, as a member of the microRNAs, miR-19b cooperatively regulates the differentiation process from osteoblast precursor cells to osteoblasts in bone cells along with other microRNAs. These microRNAs achieve this function by targeting the transcription factor Runx2, further supporting the important role of miR-19b in bone formation [30]. WWP1 could regulate cell survival by affecting cytoskeleton and cell adhesion [31]. This finding suggests that WWP1 may indirectly affect the osteogenic differentiation process by regulating cell cytoskeleton and adhesion changes and may co-regulate bone formation with miR-19b. Based on these research results, it could be seen that miR-19b, WWP1, and related signaling pathways play important roles in regulating osteogenic differentiation. The findings of this study indicate that loading EVs into GEL-OCS/MBGN biomimetic composite hydrogel could successfully regulate the miR-19b/WWP1 axis, thus promoting the repair of rat bone defects. It provides new insights for future research and bone defect repair and regeneration applications. It is expected to provide more effective and safer technical means for treating bone defects.

In animal experiments conducted in vivo, Gel-OCS/MBGN@EVs have been proven to regulate the miR-19b/WWP1 signaling axis, significantly promoting bone repair in rats with bone defects. The study results indicated that the repair of rat bone defects could be promoted by using composite hydrogel Gel-OCS/MBGN to load EVs and regulate the miR-19b/WWP1 signaling axis. A study introduced the tunable degradation and gelation behavior, excellent mechanical properties, and osteogenic activity of Gel-OCS/MBGN hydrogels [10]. Another study showed that administering EVs packaged with miR-19b promotes neuronal growth and synaptic connectivity after spinal cord injury in rats [12]. Another study found that through the EVs-mediated miR-130a signaling regulation, nano-sized calcium silicate (nano-SiCa) could promote osteogenic differentiation [32]. This discovery provides a new research direction for tissue engineering and bone defect treatment. It provides important evidence for further exploration of the mechanism of action of EVs in bone tissue repair. In addition, the study also provides new ideas and approaches for the development of novel materials for the treatment of bone defects.

**Fig. 10:**
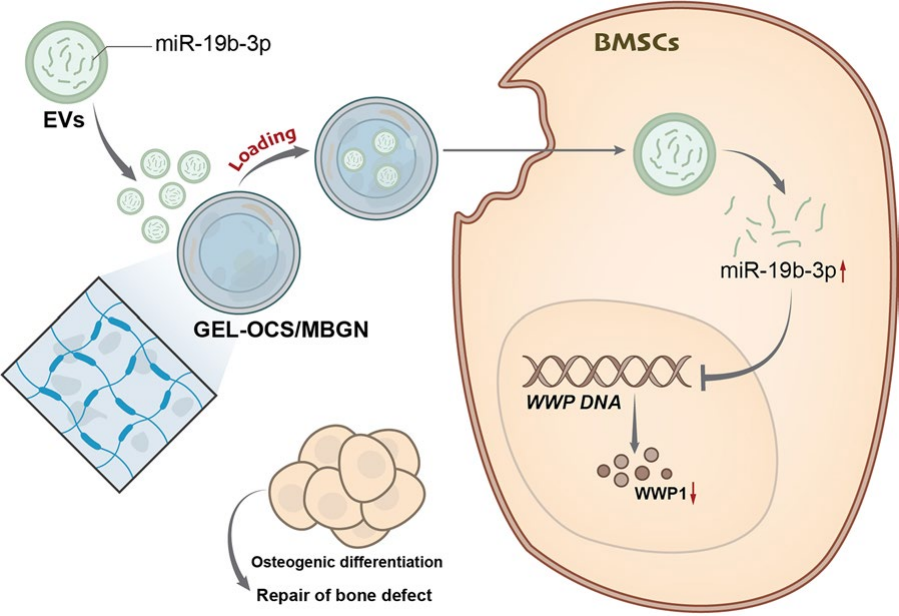
Mechanism diagram of biomimetic composite hydrogel-loaded extracellular vesicles mediating the regulation of osteogenic differentiation through the miR-19b-3p/WWP1 signaling pathway in the repair of rat bone defects

In summary, functional Gel-OCS/MBGN@EVs were obtained by constructing a composite hydrogel of GEL-OCS/MBGN and loading EVs onto it. EVs could deliver miR-19b-3p to BMSCs, inhibit the expression of WWP1, and promote the osteogenic differentiation of BMSCs, ultimately facilitating bone regeneration in rat bone defects (Fig. 10). This study effectively connected EVs and bone defects by constructing Gel-OCS/MBGN and loading EVs derived from BMSCs. The miR-19b-3p/WWP1 axis was utilized to regulate the osteogenic differentiation process of BMSCs, ultimately promoting new bone formation in rats with bone defects. The research results indicate that Gel-OCS/MBGN@EVs could promote the differentiation of BMSCs into osteoblasts by regulating the expression of WWP1 through miR-19b-3p transmission and ultimately play a good role in the rat bone defect repair model. The innovation of this study lies in effectively connecting EVs with bone defects by constructing Gel-OCS/MBGN@EVs and promoting bone defect repair in this way. In addition, the study also revealed the importance of the miR-19b-3p/WWP1 axis in the bone defect repair process and provided an important theoretical basis for further research in this field.

However, this study also has some limitations. First, the study was conducted only on mice and in vitro cells, and further experimental studies are required to validate whether these results could be translated to humans. Secondly, the study did not investigate the safety and efficacy of Gel-OCS/MBGN@EVs after long-term use. Finally, this study only investigates the role of the miR-19b/WWP1 signaling axis in osteogenic differentiation. At the same time, other signaling pathways and factors may also play a role, which requires further research exploration. Future research should further strengthen the study on EVs, explore the detailed mechanism of how EVs promote osteogenic signal transduction, improve the research on miR-19b-3p/WWP1 axis in the bone defect repair process, and further expand the sample size and strengthen long-term effect evaluation and side effect evaluation. These efforts will further promote the application of biomimetic composite hydrogels in bone defect repair, providing better options for clinical treatment.

## Materials and methods

### Cell culture and identification

Cell culture: Rat BMSCs (CM-R131, Procell) were cultured in α-MEM medium (SH30265.01, HyClone, Thermo Fisher Scientific, USA) supplemented with 15% fetal bovine serum (FBS; 10091148, Thermo Fisher Scientific, USA) and 100 U/mL penicillin–streptomycin solution (10378016, Thermo Fisher Scientific, USA), and maintained at 37 °C and 5% CO_2_ in a culture incubator. When the confluence rate of BMSCs reaches 80%, passaging should be conducted. The 3rd generation BMSCs were used for differentiation detection and extraction of EVs [33]. BMSCs identification: After washing with PBS (E607008-0500, Sangon Biotech, China), a single-cell suspension was prepared at a concentration of 1 × 10^6^/mL. Grouped cells were incubated with fluorescent-tagged antibodies: CD44-FTITC (ab30405, Abcam), CD90-PE (ab24904, Abcam), CD45-FITC (ab210220, Abcam), CD34-PE (ab23830, Abcam) and IgG (ab172730, Abcam) at 4 °C for 30 min. Subsequently, unbound antibodies were washed off with PBS, and the expression of corresponding labeled antibodies in the sample was analyzed using a flow cytometer. According to the instructions of the reagent kit (PD-003/4/5, Procell) for inducing differentiation (osteogenic, adipogenic, and chondrogenic) of BMSCs, the osteogenic, adipogenic, and chondrogenic differentiation ability of BMSCs was observed by staining with Alizarin Red S (ARS), Oil Red O and Alcian Blue respectively [34].

### Cell transfection

According to the sequences of miR-19b-3p and WWP1 published by NCBI, Shanghai Sangon Biotech was commissioned to construct the following plasmids: oe-NC, oe-WWP1, NC mimic, miR-19b-3p mimic, NC inhibitor, and miR-19b-3p inhibitor. The gene vectors for overexpression and silence were constructed using the following plasmids: pGPU6/Neo (Genomeditech, Shanghai, China) for silence and pCMV6-AC-GFP (FunGenome, Hunan, China) for overexpression. Digest BMSCs cells with trypsin first, then inoculate 4 × 10^5^ cells per well into a 6-well plate and cultivate them to form a monolayer. Next, remove the culture medium and transfect according to the Lipofectamine 2000 manual (11668-019, Invitrogen, New York, California, USA). After transfection, the cells were cultured at 37 °C under 5% CO_2_ for 6–8 h, then changed the complete culture medium and incubated for another 48 h to extract RNA and protein for subsequent experiments [35].

### Isolation, purification, and identification of EVs derived from BMSCs

Method for isolation of EVs derived from BMSCs: BMSCs transfected with inhibitor NC and miR-19b-3p inhibitor were cultured until 80–90% confluence. Remove the supernatant, wash twice with PBS, replace with FBS culture medium containing 10% EVs depletion, and continue to culture for 48 h in a 37 °C CO2 incubator. Then, the collected supernatant would be centrifuged stepwise. Firstly, centrifuge at 500*g* for 15 min at 4 °C to remove the cellular debris. Then, centrifuge at 2000*g* for 15 min at 4 °C to remove apoptotic bodies or cellular debris. Finally, centrifuge at 10,000*g* for 20 min at 4 °C to remove large vesicles. Next, filter through a 0.22 μm filter and remove EVs by ultracentrifugation at 110,000*g* for 70 min at 4 °C. After resuspending the remaining material, use 100 μL of sterile PBS for downstream experiments. Beckman ultracentrifuge (Optima L-90K, bio-thing) was used for the high-speed centrifugation steps, while Beckman Allegra X-15R benchtop centrifuge (Beckmancoulter) [11] was used for the remaining low-speed centrifugation steps.

NanoSight nanoparticle tracking analysis (NTA): 20 μg of EVs were dissolved in 1 mL of PBS, vortexed for 1 min, and measured using a NanoSight nanoparticle tracking analyzer (Malvern Instruments Ltd, Malvern Panalytical) and the corresponding software Zetaview 8.04.02. Calibrate using 110 nm polystyrene particles. The temperature is maintained at 27.65 °C to directly observe and measure the size distribution of EVs [11].

Transmission electron microscopy (TEM): 20 μL of a freshly prepared sample of ultracentrifuged EVs were loaded onto carbon-coated copper electron microscopy grids, allowed to stand for 2 min, and then negatively stained with ammonium molybdate (Sigma-Aldrich, USA, 12501-23-4) for 5 min. The grids were washed thrice with PBS to remove excess ammonium molybdate, then air-dried on filter paper until partially dry. The image was acquired with a Hitachi H7650 transmission electron microscope (DOLEE observation) at an accelerating voltage of 80 kV [36].

Western blot technique was used to identify the surface markers of EVs: EVs suspension was concentrated, and its protein content was measured using a BCA assay kit (Thermo Fisher Scientific, USA, 23227). The SDS-PAGE gel was prepared, and the proteins were denatured and electrophoresed. Then, the EVs-specific marker proteins HSP70 (ab2787, Abcam, USA, 1:1000), CD9 (SBI, USA, EXOAB-CD9A-1, 1:1000), CD81 (SBI, USA, EXOABCD81A-1, 1:1000), as well as the endoplasmic reticulum marker protein Calnexin (Abcam, ab133615, 1:1000) were detected after transfer [36].

EV labeling method: DiR (Thermo Fisher Scientific, D12731) was added to the EV solution at a concentration of 1:400 and incubated for 30 min. The solution was then ultracentrifuged at 100,000*g* for 90 min to remove excess dye and obtain DiR-labeled EVs [36].

### Gel-OCS/MBGN hydrogel synthesis reagents and materials

A biomimetic composite hydrogel was prepared by uniformly mixing OCS/MBGN mixture and gelatin (type B, with a gel strength of about 100 g Bloom, G108398-500 g, Cas(9000-70-8), Aladdin reagent, including white gelatin, animal gelatin, sinew glue, silver gelatin, Gelatin(Aladdin-e.com)) at a volume ratio of 1:1 under a temperature of 37 °C. OCS is prepared from sodium chondroitin sulfate A (CAS 39455-18-0, Aladdin Reagent, CSA, Chondroitin 4-sulfate sodium salt (Aladdin-e.com)) and sodium periodate (AR, ≥ 99.5%, S104090-500 g, CAS 7790-28-5, Aladdin Reagent, sodium periodate, meta-periodate, and sodium meta periodate (Aladdin-e.com)). MBGN was prepared by mixing tetraethyl orthosilicate (TEOS, AR, 98%, Sigma-Aldrich), cetyltrimethylammonium bromide (CTAB, AR, ≥ 99%, 30037416, Shanghai State-owned Assets Chemical Reagent Co., Ltd.), calcium nitrate (CN, AR 99%, Tianjin Fuchen Chemical Reagent Factory), and ammonia (AR, 25–28%, A112077-500 ml, Cas(1336-21-6), Aladdin Reagent, ammonium hydroxide solution) together [10].

### Synthesis of oxidized chondroitin sulfate (OCS)

Dissolve 1.25 g of CS in 20 mL of distilled water and stir at 4 degrees Celsius. After the complete dissolution of CS, add 1.93 g of sodium periodate to the solution and react for 6 h in dark conditions. The molar ratio of CS to sodium periodate is 1:1 for oxidation. Finally, transfer the above solution into a dialysis bag with a molecular weight cutoff of 3500 and dialyze in 2 L of distilled water at room temperature for 24 h. Change the water every 6 h. Afterward, pour the dialysis solution into a 50 mL centrifuge tube, freeze it at − 20 °C for 24 h, then use an FD-10 freeze dryer (made in China) for freeze drying for 7 days to obtain OCS powder [10].

### The synthesis of MBGN

Dissolve 2.80 g CTAB in 132 mL deionized water under stirring at 35 °C. After completely dissolving CTAB, add 40 mL of EA and continue stirring for 30 min. Then add 28 mL of ammonia solution (1 mol/L) and stir for 15 min. Then add 14.40 mL TEOS and stir for 30 min; finally, add 6.52 g CN. Further, stir the above mixture for 4 h. Due to the formation of colloids, the mixture gradually turns into a milky white color. Collect colloid particles by centrifugation at 8000 rad/s, wash them three times with water, and then wash them with ethanol. Dry the collected sediment at 60 °C for 24 h, then grind it into a fine powder using a mortar. Finally, heat the powder to 700 °C and maintain it for 3 h to remove organic matter and nitrates to obtain MBGNs [10].

### Preparation of composite Gel-OCS/MBGNs hydrogels

Dissolve OCS powder in 0.05 M borate buffer solution to prepare a 10% (w/v) OCS solution. Subsequently, ultrasound dispersion was conducted at room temperature for 10 min, and various concentrations of MBGNs were added to the OCS solution to prepare MBGNs-contained OCS solutions. Heat gelatin to 60 °C to dissolve and prepare a 30% (w/v) gelatin solution. Next, mix the OCS/MBGN mixture with gelatin solution in a 1:1 volume ratio, and then pour into the mold to form a hybrid gel-OCS/MBGN hydrogel at 37 °C. The final concentration of MBGNs in the resulting hydrogels was 0% and 15% (w/w) [10].

### OCS characterization

Dissolve CS and OCS separately in deuterium oxide (D2O, 99.9% purity, Adamasβ) for nuclear magnetic resonance (NMR) analysis (AVANCE III HD 400, Bruker) and use tetramethylsilane (TMS) as an internal standard at 25 °C. The chemical structure and functional groups of MBGNs and OCS could be detected with higher resolution (4 cm^−1^) using Fourier transform infrared spectroscopy (FTIR) in transmission mode, with 16 scans taken in the wavelength range of 400 to 4000 cm^−1^, using a Nicolet 6700 instrument from Thermo Fisher, USA [10].

### Field emission scanning electron microscope (FE-SEM)

We used the FE-SEM (Ultra 55) from the German company Carl Zeiss AG to investigate the morphology of MBGNs and Gel-OCS-MBGN composites. Before characterization, we dispersed the MBGNs in ethanol and then dropped them onto a silicon wafer. Before observing SEM, we freeze-dried the hydrogel sample for three days. We used gold (SC7620) from Quorum Technologies in the UK for a 60-s sputter coating. During SEM observation, we examined samples of MBGNs and Gel OCS/MBGN composite hydrogels by energy-dispersive spectroscopy (EDS) [10].

### X-ray diffraction (XRD) and Fourier transform infrared spectroscopy (FTIR)

Under a generator voltage of 40 kV and a tube current of 40 mA, the mineralized layer of MBGNs and hydrogels was analyzed using XRD (Ultima III, Japan). The scanning speed is 2°/min, and the 2θ range is between 10° and 80°. Using FTIR in transmission mode, the composite hydrogel's chemical structure and functional groups were detected with a resolution of 4 cm^−1^ and 16 scans (wavelength range 500–4000 cm^−1^). Before analyzing the gel-OCS/MBGN hydrogel, it must first be freeze-dried [10].

### Rheology and dynamic mechanical, and thermal analysis

Firstly, the gelation time of the hydrogel is determined by recording the storage modulus and loss modulus (elastic modulus) through an oscillatory time scan experiment performed by setting the oscillation frequency to 1 Hz and applying a 5% shear strain. Use Anton Paar's rotational rheometer (Physica MCR301) to evaluate the rheological properties of mixed hydrogels. Place the cross-linked hydrogel (Φ25 mm × 2 mm) on the sample stage and perform the test using a 25 mm diameter plate device at 37 °C by obtaining the storage modulus (G') and the loss modulus (G'') in the range of 0.1 and 100 rad/s from the frequency-modulus curve, with a strain amplitude of 5.0%. In addition, the compressive strength of the saturated hybrid hydrogel was studied using Dynamic Mechanical Analysis (DMA, TA Instruments, Q800) at a pressure rate of 3 N/min and 25 °C. Finally, the compressive modulus was calculated in the DMA test, using the stress–strain curve's linear region to evaluate the cross-linked hydrogel's compressive modulus (Φ10 mm × 5 mm) [10].

### Extracellular mineralization and degradation

Soak the cross-linked hydrogel (Φ8 mm × 2 mm) in 10 mL SBF in a centrifuge tube at 37 °C. Change the SBF every other day. After incubation for 7 days, remove the sample from SBF and rinse it with deionized water to eliminate excess SBF. As described above, the samples were characterized by SEM–EDS, FTIR, and XRD before undergoing freeze-drying treatment. Measure the mass loss of the hydrogel in PBS (pH 7.4) to evaluate it’s in vitro degradation. Add about 1 mL of hydrogel to 3 mL of PBS at 37 °C. Remove PBS and record the quality daily to evaluate the degradation of the hydrogel. The following equation could calculate the percentage of quality loss (%): quality loss (%) = (M1-M2)/M1 * 100%, where M1 and M2 represent the quality of the hydrogel before and after soaking in PBS, respectively. Meanwhile, the pH value of PBS-containing water gel was also measured for changes [10].

### Gel-OCS/MBGNs hydrogel loaded with EVs

Mix the prepared Gel-OCS/MBGNs hydrogel with EVs isolated from BMSCs labeled with DiR (D12731, Thermo Fisher Scientific), place the mixture in an ice bath until dissolved, and store the mixture at 4 °C. Place the cells in a culture incubator containing 5% CO_2_ and incubate in the dark for 12 h. Prepare a 1:1000 DAPI nuclear staining buffer, wash cells with PBS three times, place cells on ice, and incubate in the dark for 30 min [37]. Four images with different fields of view were randomly selected under an optical microscope (Leica®, Germany) for statistical analysis.

### The in vitro release of EVs in Gel-OCS/MBGNs@EVs

Soak the Gel-OCS/MBGNs@EVs in a 15 mL centrifuge tube containing 4 mL of PBS. At different time points (1, 3, 6, 9, 12, 15, 18, and 21 days), 100 μL of supernatant was replaced with 100 μL of fresh PBS. The concentration of EVs in the supernatant was evaluated by detecting the fluorescence intensity of DiR (Ex: 754 nm, Em: 778 nm) in the supernatant. Draw release curve, determined by the ratio of supernatant to total fluorescence intensity [38].

### CCK-8 and dead cell apoptosis kit are used to evaluate cell viability and proliferation

The live/dead staining experiment for cell viability was performed using the Live/Dead assay kit (L10119, Invitrogen, USA) following the manufacturer's instructions. BMSCs were seeded at a density of 2 × 10^4^ cells/well into 48-well plates containing different hydrogels. Prepare a staining solution with a concentration of 2 μM Calcein-AM and 4 μM ethidium homodimer-1, and add it to the wells—culture the cells in a CO_2_ incubator at 37 °C and 5% CO2 for 30 min. Imaging of live cells (green, Ex: 480 nm, Em: 530 nm) and dead cells (red, Ex: 530 nm, Em: 645 nm) was performed using an inverted fluorescence microscope (Olympus IMT-2/).

The cell proliferation experiment was conducted using CCK-8 reagent (CA1210, Solarbio) according to the following steps. BMSCs were seeded at a density of 1 × 10^4^ cells/well in 48-well plates containing different hydrogels. On the first, third, fifth, and seventh days of cultivation, the fresh culture medium was added to the 48-well plate with 10% CCK8 reagent and incubated for 1 h under dark conditions. Afterward, transfer 100 μL of the supernatant to a 96-well plate for absorbance measurement at 450 nm using a microplate spectrophotometer (Bio-Tek, UK) [38].

The cell adhesion experiment was performed using iFluor™ 488-phalloidin (ab176753, Abcam) and DAPI (ab285390, Abcam) for immunofluorescence staining. BMSCs were seeded at a density of 1 × 10^5^ cells/well into 35 mm culture dishes with different hydrogels. Fix cells in 4% paraformaldehyde and permeabilize with PBS buffer containing 1% Triton X-100. Afterward, stain with phalloidin solution for 1 h at room temperature, followed by staining with DAPI solution for 5 min. Finally, cells were imaged using a laser scanning confocal microscope (Olympus FV1000, BX61W1).

### Induction and identification of osteoblast differentiation

Osteoblast differentiation induction: BMSCs were 3D cultured in hydrogel supplemented with 10 mM β-glycerophosphate, 100 nM dexamethasone, and 50 mg/mL ascorbic acid-2-phosphate in the culture medium to obtain osteogenic induction medium. In the process of BMSC osteogenic differentiation, the culture medium is replaced every three days. Use GelMA digestion kit (EFL-GM-LS-001, Cellendes) and trypsin (R001100, Thermo Fisher Scientific) to degrade the hydrogel for cell retrieval.

Alkaline phosphatase (ALP) staining: ALP staining was performed using the alkaline phosphatase staining kit (40749ES60, Yeasen, China) following the manufacturer's instructions. After induced for 7 days, fix the BMSCs in 4% paraformaldehyde, washed with PBS, and stain for 30 min. Observe stained cells using a microscope (IX73, OLYMPUS, Japan). To evaluate the ALP activity of BMSCs, the ALP activity assay kit (MAK411, Sigma-Aldrich) was used to characterize the cell lysate. The alkaline phosphatase activity was measured by incubating with a dinitrophenylphosphate solution and then measuring the absorbance at 520 nm with a microplate spectrophotometer (Bio-Tek, Thermo Fisher Scientific).

Alizarin Red S (ARS) staining: staining of the calcium deposited in BMSCs induced for 21 days with ARS. Fix BMSCs with 4% formaldehyde, stain them with ARS solution (pH 1.354, PHYGENE) at room temperature for 30 min, and wash them with PBS. Observe stained cells using an inverted microscope (IX73, OLYMPUS). Calcium deposits stained with 10% cetylpyridinium chloride (CPC, C0732, Sigma-Aldrich) were dissolved, and the mineralization process was measured using a microplate spectrophotometer at 562 nm by determining absorbance [38].

### Immunofluorescence co-staining

Fix cells with 4% formaldehyde at room temperature for 15 min, followed by two washes with PBS. Next, cells were treated with 0.5% Triton X-100 (P0096, Beyotime) for 10 min to increase cellular permeability. Then incubate overnight at 4 °C with primary anti-OPN rabbit antibody (ab11503, 1:200, Abcam) and react with it. After incubation, wash the slices three times with PBS solution, then incubate with secondary antibody (ab150129/ab150077, 1:200, Abcam) conjugated with Alexa Fluor 488 for 1 h. Then, wash the sections three times with PBS and stain the cells with 10 μg/mL DAPI (D3571, Thermo Fisher, USA) at room temperature [39]. Finally, save the slices at 4 °C and observe the cells using a fluorescence microscope (IMT-2, Olympus).

### Transcriptome sequencing

This study was approved by Institutional Animal Ethics Committee of The Third Hospital of Hebei Medical University. Total RNA was extracted from three samples of rat BMSCs and three osteoblast cells (OB) using TRI reagent (Sigma, USA) following the manufacturer's instructions. The purity and concentration of DNA were then measured using a Nanodrop 2000 spectrophotometer (DeNovix, USA) and Qubit 2.0 fluorometer with the Quant-IT dsDNA HS assay kit (Thermo Fisher Scientific). Messenger RNA is extracted from total RNA and purified using oligo-dT magnetic beads. 1 μg DNA was sheared into 250-kb fragments by ultrasonic treatment, followed by end repair, dA addition, and ligation to indexed Illumina sequencing adapters. Partial enrichment was performed using DNA capture probes (NimbleGen, US), and deep sequencing was conducted on the Illumina NexSeq CN500 platform. The library was checked using Qubit 2.0 and real-time polymerase chain reaction (PCR), and size distribution were analyzed by a biophysical analyzer. The raw data (in fastq format) is first processed by an internal perl script, which removes reads containing adapters, poly-N sequences, and low-quality reads, ensuring clean data. All downstream analysis is based on high-quality data. The reference genome was indexed using Hisat2 v2.0.5, and the paired-end clean reads were aligned to the reference genome. Calculate the read counts mapped to each gene using featureCounts v1.5.0-p3, and then calculate the fragments per kilobase of transcript per million mapped fragments (FPKM) of each gene based on its length and mapped read counts [40, 41].

### Differential expression analysis

We conducted differential expression analysis of sequencing data using the "limma" package in R language. We extracted differentially expressed genes under the condition that |log2FC| > 2 and ad.P.Val < 0.05. We used R language to draw heatmaps and volcano plots of differentially expressed genes [42–44].

### GO and KEGG functional enrichment analysis

Perform functional enrichment analysis on differentially expressed genes using the ClusterProfiler R package. This software package could be used for gene ontology (GO) functional enrichment analysis, covering three levels of biological process (BP), cellular component (CC), and molecular function (MF) [42]. In addition, we used the Enrichr website for KEGG analysis of genes, with a significance threshold of P < 0.05.

### Lasso regression is used to screen for feature genes

The "glmnet" package in R language is used to perform regression analysis on differentially expressed genes in sequencing data to identify the core feature genes for osteogenic differentiation of BMSCs [45].

### Dual-luciferase reporter assay

By using TargetScan analysis, potential binding sites between miR-19b-3p and WWP1 were predicted. Insert the 3'-UTR sequence of WWP1 (wild type, WT) into the pGL3-Basic vector (Promega) to construct the recombinant vector WWP1-WT. Further, the miR-19b-3p binding site mutation sequence of WWP1 was cloned into the multiple cloning site region of the pGL3-Basic vector (Promega) to generate the recombinant vector WWP1-MUT. The Dual-Luciferase Reporter Assay System kit (Promega, USA) performed transfection experiments for the wild-type and mutant luciferase reporter plasmids. Cells were transfected with mimic NC and miR-19b-3p mimic separately and harvested 48 h post-transfection. Cell lysates were collected and luciferase activity was measured with Luminometer TD-20/20 Detector (Model: E5311, Promega, USA). Relative fluorescence values were obtained by dividing RLU values measured by Renilla luciferase using firefly luciferase as an internal control [46]. The experiment was repeated 3 times in total.

### RT-qPCR

Total cellular RNA was extracted using Trizol (16096020, Invitrogen, USA). The absorbance of the solution at 260 and 280 nm was measured using spectrophotometry to evaluate the purity and concentration of the obtained RNA. The A260/A280 ratio of the sample should be ≥ 1.8. The reverse transcription kit (11483188001, Roche, Switzerland) was used to reverse transcribe mRNA, and cDNA was prepared. For miRNA, a PolyA tailing kit (B532451, Sangon Biotech Co., Ltd., China) was used to obtain cDNA with PolyA-tailed miRNA. The reverse transcription reaction was performed at 42 °C for 15 min, followed by a 5-s inactivation reaction of the reverse transcriptase at 85 °C. The reverse transcribed cDNA was diluted to 50 ng/μL and subsequently used for fluorescence quantitative PCR analysis. PCR was performed using LightCycler 480 SYBR Green I Master. The reaction conditions were as follows: initial denaturation at 95 °C for 10 min, amplification at 95 °C for 15 s, 60 °C for 20 s, 72 °C for 20 s, for 40 cycles. Using GAPDH as the internal reference for mRNA, U6 as the internal reference for miRNA, and miR16 as the internal reference for miRNA in EVs [47]. Calculate 2-ΔΔCt to represent the target gene expression fold change between experimental and control groups. The primer sequences are shown in Additional file 2: Table S2. Each experiment was repeated three times.

### Western blot

Total protein was extracted from tissues or cells using high-efficiency RIPA lysis buffer (C0481, Sigma-Aldrich Aldrich, USA) containing 1% protease inhibitor and 1% phosphatase inhibitor (ST019-5 mg, Beyotime, Shanghai, China). After being lysed at 4 °C for 15 min, the lysate was centrifuged at 15,000 r/min for 15 min. The supernatant was collected, and the protein concentration of each sample was measured using a BCA assay kit (23,227, TH&Ermo, USA). Quantify the sample by adding 5 times of loading buffer (P0015, Bi Yun Tian, China) at different concentrations, then separate the protein by polyacrylamide gel electrophoresis and transfer it to a PVDF membrane (IPVH00010, Millipore, Billerica, MA, USA). Block with 5% BSA at room temperature for 1 h. Incubate with the first antibody at 4 °C overnight. On the second day, wash the membrane thrice using TBST for 5 min each time. Then, incubate with a diluted solution of goat anti-rabbit IgG (1:2000, ab205718, Abcam, UK) or goat anti-mouse IgG (1:2000, ab6789, Abcam, UK) labeled with HRP at room temperature for 1.5 h. After washing the TBST membrane three times for five minutes each, add the developing solution (NCI4106, Pierce, Rockford, IL, USA) and carry out the development process. Protein quantification analysis was performed using ImageJ software. The grayscale values of each protein were compared to the grayscale ratio of the internal control GAPDH, and each experiment was repeated three times [48].

### Constructing rat models with femoral bone defects

We purchased 24 SD male rats (7 weeks old, weighing 250–300 g) with strain code 101 from Beijing Vital River Laboratory Animal Technology Co., Ltd. These rats were raised in an SPF-grade animal laboratory with a humidity of 60% to 65%, a temperature of 22–25 °C, and free access to food and water under a 12-h light–dark cycle. After one week of adaptive feeding, we observed the health conditions of the rats before starting the experiment. This experiment has been approved by the Animal Ethics Committee of The Third Hospital of Hebei Medical University and conforms to the principles for the management and use of experimental animals in the local area.

When performing the femoral defect surgery, we performed general anesthesia on each rat. We made a 1.5 cm longitudinal incision in the center of the palpable bone protrusion on the outer side of the femoral condyle of each leg while taking sufficient sterile precautions. Then we carefully dissected the subcutaneous tissue, fascia, muscles, and periosteum, exposing the underlying bone. We induce bone defects using an oral micromotor to form a 4 mm deep hole in the subchondral bone below with a 4 mm annular bone drill. Next, we will implant corresponding hydrogels into the defect area, followed by exposure to 405 nm light for 90 s (25 mW/cm^2^) to form a secondary network. Finally, we carefully layered and sutured the soft tissues and skin. After 8 weeks, we euthanized rats to obtain femurs. We fixed it using over 4% formaldehyde solution (24 h) and preserved it in 75% ethanol for subsequent analysis [38, 49, 50].

We randomly divided the rats into 4 groups, with 6 rats in each group: (1) PBS group; (2) Gel-OCS/MBGNs@EVs group (injection of Gel-OCS/MBGN@EVs hydrogel); (3) Gel-OCS/MBGNs@EVs-inhibitor NC group (transfecting inhibitor NC obtained EVs into BMSCs, and Gel-OCS/MBGNs@EVs-inhibitor NC hydrogel was prepared for injection); (4) Gel-OCS/MBGNs@EVs-miR-19b-3p inhibitor group (EVs were obtained by transfecting miR-19b-3p inhibitor into BMSCs, and Gel-OCS/MBGNs@EVs-miR-19b-3p inhibitor hydrogel was prepared for injection). Inject 100 μL water gel per group, and the final concentration of EVs is 0.2 μg/μL.

### Micro-CT and X-ray

Bruker's Micro-CT used a source current of 280 µA, a source voltage of 90 kV, and an exposure time of 550 ms. Each defect region was scanned and analyzed in sagittal and axial planes with the same calibration parameters and reconstructed using NRecon software. To quantify the newly mineralized tissue, bone mineral content (BMC) and new bone volume (BV/TV%) were calculated. After the specimen was taken, X-ray images were also taken [38, 50, 51].

### H&E staining and Masson staining

After decalcification in ethylenediaminetetraacetic acid (EDTA) for 6 weeks, the femur tissue was dehydrated and embedded in paraffin through a graded ethanol series. After processing, cut it into 6 μm thick slices for staining. According to the instructions, use Su Mu Jing & Yi Hong (H&E, G1076-500ML, Servicebio) and Masson's trichrome staining kit (G1340, Solarbio, Beijing, China) to stain the slices [38, 39, 52].

### Statistical analysis

Data statistical analysis required for this study was conducted using the SPSS 21.0 software produced by IBM. The mean ± standard deviation represents the measurement data. Firstly, normality and homoscedasticity tests are conducted. Suppose the data fit the normal distribution and has equal variances. In that case, the t-test is used to compare two groups of data, and a one-way analysis of variance is used to compare multiple data groups. Tukey's method is used for post-hoc tests. When comparing different data groups at different time points, repeated measures analysis of variance is adopted, and Tukey's method is used for the post-hoc test. When the P value is less than 0.05, it indicates that the difference is statistically significant.

### Supplementary Information

**Additional file 1: Fig. S1.** Characterization of BMSCs and EVs. Note: (A) Observe the morphology of BMSCs with an inverted microscope, scale bar = 100 μm; (B) Detect the osteogenic, adipogenic, and chondrogenic differentiation ability of BMSCs by staining with Alizarin Red, Oil Red O, and Alcian Blue, scale bar = 100 μm / 50 μm; (C) Detect the expression of BMSCs markers by flow cytometry; (D) Observe the morphology characteristics of EVs with TEM image, scale bar = 200 μm; (E) Detect the size of EVs by NTA; (F) Detect the expression of EVs markers by Western blot. The experiment should be repeated at least three times.

**Additional file 2.** Primer sequences of RT-qPCR, Top 10 KEGG enrichment analysis entries.

## Data Availability

The datasets generated and/or analyzed during the current study are available from the corresponding author on reasonable request.
