# Supplementary figures and images for: Biomimetic composite hydrogel promotes new bone formation in rat bone defects through regulation of miR-19b-3p/WWP1 axis by loaded extracellular vesicles
Source: J Nanobiotechnology. 2023 Nov 30;21:459. doi: 10.1186/s12951-023-02201-w (PMC10691144; doi:10.1186/s12951-023-02201-w)

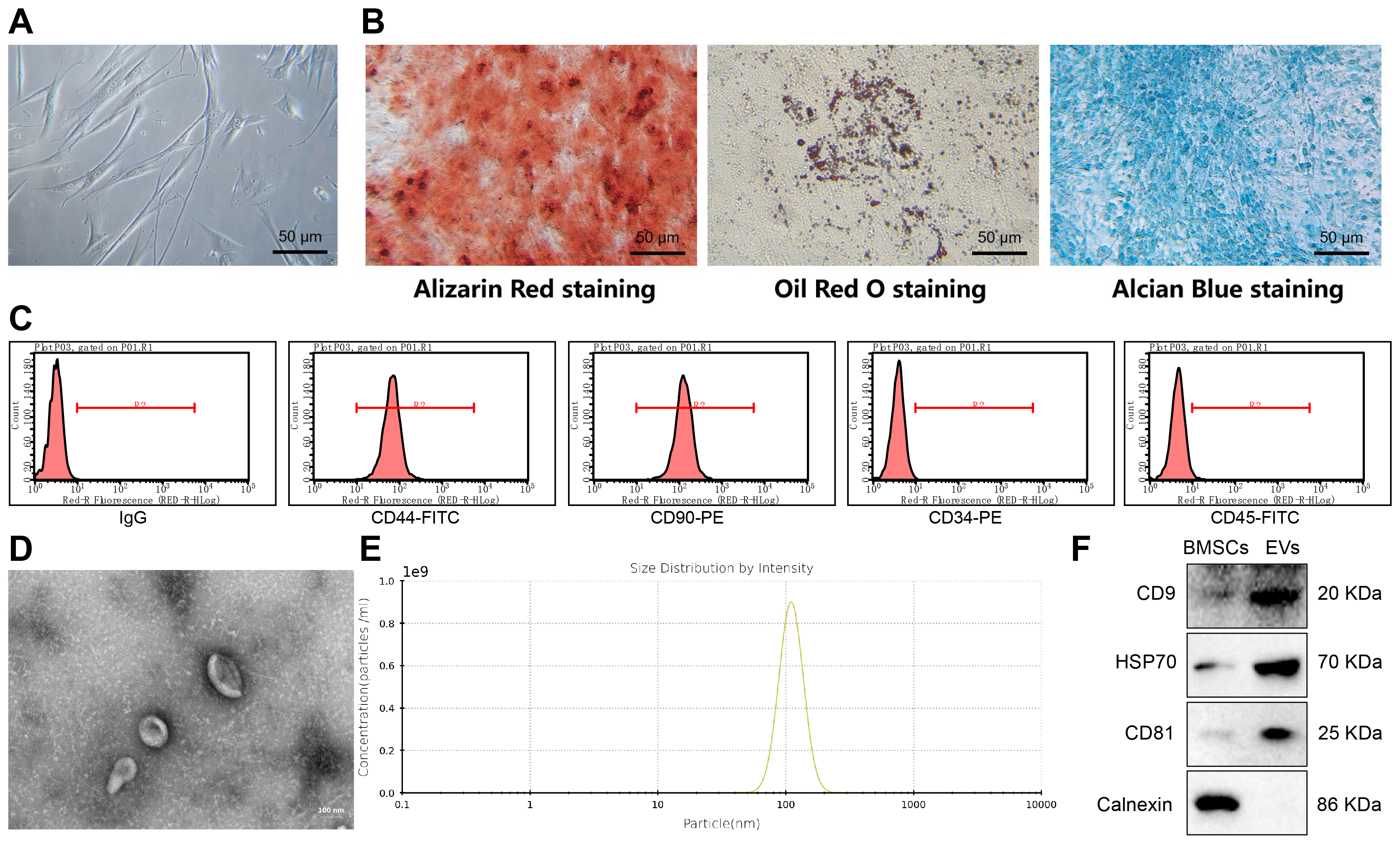

Supplement: Supplementary file 1 — Additional file 1: Fig. S1. Characterization of BMSCs and EVs. Note: (A) Observe the morphology of BMSCs with an inverted microscope, scale bar = 100 μm; (B) Detect the osteogenic, adipogenic, and chondrogenic differentiation ability of BMSCs by staining with Alizarin Red, Oil Red O, and Alcian Blue, scale bar = 100 μm / 50 μm; (C) Detect the expression of BMSCs markers by flow cytometry; (D) Observe the morphology characteristics of EVs with TEM image, scale bar = 200 μm; (E) Detect the size of EVs by NTA; (F) Detect the expression of EVs markers by Western blot. The experiment should be repeated at least three times. [file 12951_2023_2201_MOESM1_ESM.jpg]
