# Supplementary material for: Biomimetic composite hydrogel promotes new bone formation in rat bone defects through regulation of miR-19b-3p/WWP1 axis by loaded extracellular vesicles
Source: J Nanobiotechnology. 2023 Nov 30;21:459. doi: 10.1186/s12951-023-02201-w (PMC10691144; doi:10.1186/s12951-023-02201-w)
Supplement: Supplementary file 2 — Additional file 2. Primer sequences of RT-qPCR, Top 10 KEGG enrichment analysis entries. [file 12951_2023_2201_MOESM2_ESM.docx]

TableS1 Primer sequences of RT-qPCR

| Genes | Sequences |
| --- | --- |
| Runx2 (Rat) | Forward: 5’-CACAAGTGCGGTGCAAACTT-3’ |
|  | Reverse: 5’-AAGAGGCTGTTTGACGCCAT-3’ |
| Osterix (Rat) | Forward: 5’-ATTGCCAGTAATCTTCGTGCC-3’ |
|  | Reverse: 5’-TAGTGAGCTTCTTCCTGGGGA-3’ |
| Alpl (Rat) | Forward: 5’- TCCTTAGGGCCACCGCT-3’ |
|  | Reverse: 5’-GGCAGTGTCAGCCGTTAATTG-3’ |
| Opn (Rat) | Forward: 5’-CCAGCCAAGGACCAACTACA-3’ |
|  | Reverse: 5’-CTGCCAAACTCAGCCACTTTC-3’ |
| Ocn (Rat) | Forward: 5’-CTAGCGGACCACATTGGCTT-3’ |
|  | Reverse: 5’-AGCTGTGCCGTCCATACTTT-3’ |
| WWP1 (Rat) | Forward: 5’-TCCTGGTACACAAATCAAGGTGA-3’ |
|  | Reverse: 5’-ACTGTAACCTTCCACTGTGGTT-3’ |
| miR-19b-3p (Rat) | Forward: 5’-TGTGCAAATCCATGCAAAACTGA-3’ |
|  | Reverse: Inverse Universal Primers |
| U6 (Rat) | Forward: 5’-GGGAGGGAGGGCATACTTTC-3’ |
|  | Reverse: Inverse Universal Primers |
| GAPDH (Rat) | Forward: 5’-TGTTCTAGAGACAGCCGCATC-3’ |
|  | Reverse: 5’-AATCCGTTCACACCGACCTT-3’ |

**Table S2. Top 10 KEGG enrichment analysis entries**

| Term | P-value | Adjusted P-value | Z Score |
| --- | --- | --- | --- |
| Cytokine-cytokine receptor interaction | 6.25308E-09 | 6.94092E-07 | -0.333333333 |
| Rheumatoid arthritis | 1.76452E-06 | 9.79311E-05 | -0.447213595 |
| Viral protein interaction with cytokine and cytokine receptor | 7.13265E-05 | 0.002639079 | 0 |
| HIF-1 signaling pathway | 9.9858E-05 | 0.002771059 | -1 |
| Malaria | 0.0001955 | 0.004340099 | 0.577350269 |
| Transcriptional misregulation in cancer | 0.000888176 | 0.015518471 | 1 |
| IL-17 signaling pathway | 0.00125321 | 0.015518471 | 0.577350269 |
| Hematopoietic cell lineage | 0.001455673 | 0.015518471 | -0.577350269 |
| AGE-RAGE signaling pathway in diabetic complications | 0.001498488 | 0.015518471 | 0.577350269 |
| Human cytomegalovirus infection | 0.001571566 | 0.015518471 | 0 |
